# Supplementary material for: Patients and healthcare workers’ preferences for using smart lockers in accessing and dispensing chronic disease medication in Nigeria: Findings from a descriptive cross-sectional study
Source: PLoS One. 2024 Jul 5;19(7):e0303625. doi: 10.1371/journal.pone.0303625 (PMC11226012; doi:10.1371/journal.pone.0303625)
Supplement: Attachment — Submitted filename: PONE-D-23-39195_R2_reviewer.pdf [file pone.0303625.s009.pdf]

PLOS ONE

Patients and Healthcare Workers' Preferences for Using Smart Lockers in Accessing and Dispensing Chronic Disease Medication in Nigeria: Findings from a Descriptive Cross-Sectional Study

--Manuscript Draft--

**Manuscript Number:**	PONE-D-23-39195R2
**Article Type:**	Research Article
**Full Title:**	Patients and Healthcare Workers' Preferences for Using Smart Lockers in Accessing and Dispensing Chronic Disease Medication in Nigeria: Findings from a Descriptive Cross-Sectional Study
**Short Title:**	The Preference for the Use of Smart Lockers for Dispensing Chronic Disease Medication in Nigeria.
**Corresponding Author:**	Ibrahim Bola Gobir, MD, MPH<br>Georgetown University Medical Center<br>Washington DC, UNITED STATES
**Keywords:**	Smart lockers, Chronic diseases, Healthcare workers, Medication, Persons Living with HIV(PLHIV)
**Abstract:**	The use of smart locker technology has been beneficial for patients with chronic diseases who require regular medication and face challenges accessing healthcare facilities due to distance, time, or mobility issues. The objective of this study was to describe patients and healthcare workers' preferences for using smart lockers for dispensing chronic disease medications in Nigeria. We employed a descriptive cross-sectional research design during our study. We focused on healthcare workers and patients with chronic diseases in 5 Nigerian states of Adamawa, Akwa Ibom, Cross River, Benue, and Niger between November and December 2021. Using a stratified random sampling technique, a semi-structured questionnaire was used to collect data from healthcare workers who provide care and treatment services to individuals with chronic diseases and persons living with one or more chronic diseases and/or their clients (participants) A total of 1,133 participants (HCWs – 405 and Patients – 728) were included in our study. The total number of responses received during the study was 1,180, and 47 (4.0%) were excluded from data analysis because they did not meet the inclusion criteria. Our findings reflect that the suitable populations to use smart lockers were HCWs and patients with HIV (HCWs – n =175, 43.2%, Patients – n=334, 45.9%) between the ages of 18 and 40 years (HCWs – 81.0%, Patients – 70.5%).There were no significant differences between the HCWs and the patients' choices on the method of orientation, i.e., they both preferred (one-on-one counselling with their healthcare providers) (p = 0.25) and notification for drug pick up (phone call) (p = 0.63). The preference for smart lockers location and the accessibility time between HCWs and patients were statistically significant (p < 0.05); the hospital or clinic as a preferred location was higher amongst patients (71.6%) than HCWs (68.9%) and the 24-hour access to smart lockers preference was lower amongst the patients (52.6%) than the HCWs (65.4%). The study findings revealed that the preferred age group that smart lockers may best serve was Persons Living with HIV (PLHIV) between 18 and 40 years old. The lockers may be placed within a secured hospital or clinic and within reach of patients in the community with 24-hour access to the smart locker for drug pick-up. The patients can be reached for medication dispensing through phone calls. The preferred method of orientation by both patients and HCWs on the use of smart lockers for dispensing medication is one-on-one counselling with their healthcare providers. Given that smart lockers have the potential to enhance medication adherence and improve health outcomes. The development of models for using smart lockers to dispense chronic disease medications to chronically ill persons in Nigeria and other populations is recommended.
**Order of Authors:**	Ibrahim Bola Gobir, MD, MPH
	Samson Agboola, MSc, PHD
	Havilah Onyinyechi Nnadozie

Powered by Editorial Manager® and ProduXion Manager® from Aries Systems Corporation

	Helen Adamu, MSc, PHD
	Fatimah Ohunene Sanni, MSc
	Aisha Adamu, MD, PHD
	Azeez Akanbi Bello
	Suzzy Angmun Otubu
	Mercy Piring'ar Niyang, MSc, MPH
Response to Reviewers:	Response to reviewer 2's comments<br>Thank you for taking the time to review our manuscript titled "Patients and Healthcare Workers' Preferences for Using Smart Lockers in Accessing and Dispensing Chronic Disease Medication in Nigeria: Findings from a Descriptive Cross-Sectional Study", submitted to PLOS ONE We appreciate your insightful comments and constructive feedback, which have greatly contributed to improving the quality of our work.<br>Reviewer 2: Thank you for this response, The title has been revised as suggested.<br>Reviewer 2: Thank you for your comment. The word "participants" has been included in the bracket.<br>Reviewer 2: We appreciate your feedback. The statement has been adjusted to sound more meaningful.<br>Reviewer 2: Your comment is appreciated, in the background of our study, the paragraphs have been adjusted.<br>Reviewer 2: Thank you for your comment. This adjustment was made during the previous revision and the subtitles merged into the background.<br>Reviewer 2: We are appreciative of your feedback. This adjustment was made during the previous revision, and the sections were merged into the background.<br>Reviewer 2: Thank you for your comment. This adjustment to the methods was made during the previous revision.<br>Reviewer 2: Thank you for your response. The discussion session has been adjusted.<br>Reviewer 2: Thank you for your suggestion on the acknowledgement. It has been adopted.
Additional Information:	
**Question**	**Response**
**Financial Disclosure**<br><br>Enter a financial disclosure statement that describes the sources of funding for the work included in this submission. Review the submission guidelines for detailed requirements. View published research articles from *PLOS ONE* for specific examples.<br><br>This statement is required for submission and **will appear in the published article** if the submission is accepted. Please make sure it is accurate.	Yes

**Funded studies**<br>Enter a statement with the following details:<br>• Initials of the authors who received each award<br>• Grant numbers awarded to each author<br>• The full name of each funder<br>• URL of each funder website<br>• Did the sponsors or funders play any role in the study design, data collection and analysis, decision to publish, or preparation of the manuscript?<br><br>Did you receive funding for this work?	
Please add funding details.<br>as follow-up to "**Financial Disclosure**<br><br>Enter a financial disclosure statement that describes the sources of funding for the work included in this submission. Review the submission guidelines for detailed requirements. View published research articles from *PLOS ONE* for specific examples.<br><br>This statement is required for submission and **will appear in the published article** if the submission is accepted. Please make sure it is accurate.<br><br>**Funded studies**<br>Enter a statement with the following details:<br>• Initials of the authors who received each award<br>• Grant numbers awarded to each author<br>• The full name of each funder<br>• URL of each funder website<br>• Did the sponsors or funders play any role in the study design, data collection and analysis, decision to publish, or preparation of the manuscript?<br><br>Did you receive funding for this work?"	-IBG<br>-GU-HRP-502 - Georgetown ICF_Smart Locker_IBG_12142021<br>-Georgetown University Medical Center Dean of Research<br>-https://cghpi.georgetown.edu/<br>-No, the funders had no role in the study design, data collection and analysis, decision to publish or preparation of the manuscript.
Please select the country of your main research funder (please select carefully as in some cases this is used in fee calculation).	NIGERIA - NG

as follow-up to "**Financial Disclosure**<br><br>Enter a financial disclosure statement that describes the sources of funding for the work included in this submission. Review the submission guidelines for detailed requirements. View published research articles from *PLOS ONE* for specific examples.<br><br>This statement is required for submission and **will appear in the published article** if the submission is accepted. Please make sure it is accurate.<br><br>**Funded studies**<br>Enter a statement with the following details:<br>• Initials of the authors who received each award<br>• Grant numbers awarded to each author<br>• The full name of each funder<br>• URL of each funder website<br>• Did the sponsors or funders play any role in the study design, data collection and analysis, decision to publish, or preparation of the manuscript?<br><br>Did you receive funding for this work?"	
**Competing Interests**<br><br>Use the instructions below to enter a competing interest statement for this submission. On behalf of all authors, disclose any competing interests that could be perceived to bias this work—acknowledging all financial support and any other relevant financial or non-financial competing interests.<br><br>This statement is required for submission and **will appear in the published article** if the submission is accepted. Please make sure it is accurate and that any funding sources listed in your Funding Information later in the submission form are also declared in your Financial Disclosure statement.	The authors have declared that no competing interests exist.

View published research articles from *PLOS ONE* for specific examples.<br><br>**NO authors have competing interests**<br><br>Enter: *The authors have declared that no competing interests exist.*<br><br>**Authors with competing interests**<br><br>Enter competing interest details beginning with this statement:<br><br>*I have read the journal's policy and the authors of this manuscript have the following competing interests: [insert competing interests here]*<br><br>* typeset	
**Ethics Statement**<br><br>Enter an ethics statement for this submission. This statement is required if the study involved:<br><br>• Human participants<br>• Human specimens or tissue<br>• Vertebrate animals or cephalopods<br>• Vertebrate embryos or tissues<br>• Field research<br><br>Write "N/A" if the submission does not require an ethics statement.<br><br>General guidance is provided below. Consult the submission guidelines for detailed instructions. **Make sure that all information entered here is included in the Methods section of the manuscript.**	National Health Research Ethics Committee of Nigeria (NHREC)<br>NHREC Approval Number NHREC/01/01/2007-16/09/2021<br>Date: 16th September, 2021<br><br>Ethical Statement<br>Informed consent<br>The survey's first page included information about the study's aims, eligibility criteria, data privacy, and researcher disclaimers. Participants provided informed consent. It was deemed sufficient to provide informed consent if a participant checked the "I agree" item on the survey. Participants' entries that did not match the inclusion criteria were not processed for data analysis. Confidentiality<br>Every entry was recorded anonymously. Participants' personally identifiable information was not collected. The subjects' information was kept private.<br>Risks and benefits<br>There were no negative effects on the subjects' rights or welfare. There were no immediate benefits to participating in the trial.<br>Ethical clearance<br>The study protocol was submitted to the Nigerian Health Research Ethics Committee (NHREC) for assessment and approval. Permission was also sought from the administrators of the hospitals where the data was collected.

**Format for specific study types**<br><br>**Human Subject Research (involving human participants and/or tissue)**<br>• Give the name of the institutional review board or ethics committee that approved the study<br>• Include the approval number and/or a statement indicating approval of this research<br>• Indicate the form of consent obtained (written/oral) or the reason that consent was not obtained (e.g. the data were analyzed anonymously)<br><br>**Animal Research (involving vertebrate animals, embryos or tissues)**<br>• Provide the name of the Institutional Animal Care and Use Committee (IACUC) or other relevant ethics board that reviewed the study protocol, and indicate whether they approved this research or granted a formal waiver of ethical approval<br>• Include an approval number if one was obtained<br>• If the study involved *non-human primates*, add *additional details* about animal welfare and steps taken to ameliorate suffering<br>• If anesthesia, euthanasia, or any kind of animal sacrifice is part of the study, include briefly which substances and/or methods were applied<br><br>**Field Research**<br><br>Include the following details if this study involves the collection of plant, animal, or other materials from a natural setting:<br>• Field permit number<br>• Name of the institution or relevant body that granted permission	
**Data Availability**<br><br>Authors are required to make all data underlying the findings described fully available, without restriction, and from the time of publication. PLOS allows rare exceptions to address legal and ethical concerns. See the PLOS Data Policy and FAQ for detailed information.	Yes - all data are fully available without restriction

A Data Availability Statement describing where the data can be found is required at submission. Your answers to this question constitute the Data Availability Statement and **will be published in the article**, if accepted.<br><br>**Important:** Stating 'data available on request from the author' is not sufficient. If your data are only available upon request, select 'No' for the first question and explain your exceptional situation in the text box.<br><br>Do the authors confirm that all data underlying the findings described in their manuscript are fully available without restriction?	
**Describe where the data may be found in full sentences. If you are copying our sample text, replace any instances of XXX with the appropriate details.**<br><br>• If the data are **held or will be held in a public repository**, include URLs, accession numbers or DOIs. If this information will only be available after acceptance, indicate this by ticking the box below. For example: *All XXX files are available from the XXX database (accession number(s) XXX, XXX.*).<br>• If the data are all contained **within the manuscript and/or Supporting Information files**, enter the following: *All relevant data are within the manuscript and its Supporting Information files.*<br>• If neither of these applies but you are able to provide **details of access elsewhere**, with or without limitations, please do so. For example:<br><br>*Data cannot be shared publicly because of [XXX]. Data are available from the XXX Institutional Data Access / Ethics Committee (contact via XXX) for researchers who meet the criteria for access to confidential data.*<br><br>*The data underlying the results presented in the study are available from (include the name of the third party*	Data set supporting the findings of this study are available within the paper and its supporting files (Supplementary data)

and contact information or URL).<br>• This text is appropriate if the data are owned by a third party and authors do not have permission to share the data.<br><br>* typeset	
Additional data availability information:	

Powered by Editorial Manager® and ProduXion Manager® from Aries Systems Corporation

Manuscript

Click here to access/download;Manuscript;Man 3_11_1_Patients and Healthcare Workers 11_3_24.docx

Patients and Healthcare Workers' Preferences for Using Smart Lockers in Accessing and Dispensing Chronic Disease Medication in Nigeria: Findings from a Descriptive Cross-Sectional Study.

Ibrahim Bola Gobir[1*], Samson Agboola[2], Havilah Onyinyechi Nnadozie[2], Helen Adamu[2], Fatimah Ohunene Sanni[2], Aisha Adamu[2], Azeez Akanbi Bello[2], Angmun Suzzy Otubo[3], Piring'ar Mercy Niyang[1]

[1]Center for Global Health Practice and Impact, Georgetown University, Washington DC, United States of America

[2]Georgetown Global Health Nigeria, Federal Capital Territory, Abuja, Nigeria

[3]Savannah Health System Innovation Limited, Federal Capital Territory, Abuja, Nigeria

[*]Corresponding author

Email: ibg7@georgetown.edu (IBG)

// Abstract

The use of smart locker technology has been beneficial for patients with chronic diseases who require regular medication and face challenges accessing healthcare facilities due to distance, time, or mobility issues. The objective of this study was to describe patients and healthcare workers' preferences for using smart lockers for dispensing chronic disease medications in Nigeria. We employed a descriptive cross-sectional research design during our study. We focused on healthcare workers and patients with chronic diseases in 5 Nigerian states of Adamawa, Akwa Ibom, Cross River, Benue, and Niger between November and December 2021. Using a stratified random sampling technique, a semi-structured questionnaire was used to collect data from healthcare workers who provide care and treatment services to individuals with chronic diseases and persons living with one or more chronic diseases and/or their clients (participants). A total of 1,133 participants (HCWs – 405 and Patients – 728) were included in our study. The total number of responses received during the study was 1,180, and 47 (4.0%) were excluded from data analysis because they did not meet the inclusion criteria. Our findings reflect that the suitable populations to use smart lockers were HCWs and patients with HIV (HCWs – n =175, 43.2%, Patients – n=334, 45.9%) between the ages of 18 and 40 years (HCWs – 81.0%, Patients – 70.5%).

There were no significant differences between the HCWs and the patients' choices on the method of orientation, i.e., they both preferred (one-on-one counselling with their healthcare providers) ($p = 0.25$) and notification for drug pick up (phone call) ($p = 0.63$). The preference for smart lockers location and the accessibility time between HCWs and patients were statistically significant ($p < 0.05$); the hospital or clinic as a preferred location was higher amongst patients (71.6%) than HCWs (68.9%) and the 24-hour access to smart lockers preference was lower amongst the patients

(52.6%) than the HCWs (65.4%). The study findings revealed that the preferred age group that smart lockers may best serve was Persons Living with HIV (PLHIV) between 18 and 40 years old. The lockers may be placed within a secured hospital or clinic and within reach of patients in the community with 24-hour access to the smart locker for drug pick-up. The patients can be reached for medication dispensing through phone calls. The preferred method of orientation by both patients and HCWs on the use of smart lockers for dispensing medication is one-on-one counselling with their healthcare providers. Given that smart lockers have the potential to enhance medication adherence and improve health outcomes. The development of models for using smart lockers to dispense chronic disease medications to chronically ill persons in Nigeria and other populations is recommended.

Keywords: Smart lockers, Chronic diseases, Healthcare workers, Medication, Persons Living with HIV(PLHIV)

Background

Chronic disease is a sickness that lasts a long time, advances slowly, is caused by genetics, the environment, or poor lifestyle choices, and requires continuous medical care [1]. Chronic diseases were responsible for more than 28 million deaths worldwide in 1990. By 2008, this number had climbed to 36 million, and 39 million in 2016 [2]. Chronic diseases remain the leading cause of death globally [3, 4]. In Nigeria alone, chronic respiratory diseases, diabetes, cancer, and cardiovascular diseases were the cause of up to 29% of all deaths in 2018. [5]. The World Health Organization (WHO) reported a 20% probability of dying prematurely from non-communicable diseases (NCDs) in Nigeria [6]. Evidence suggests that chronic disease patients experience multimorbidity because chronic diseases rarely occur in isolation. The burden of managing the treatment of chronic illnesses includes changes in lifestyle, and behaviours, regular visits to the healthcare professional or clinic, and managing medications [7]. Multimorbidity is linked to significant increases in healthcare expenses and resources due to increased primary care and specialist physician services, prescription use, emergency room visits, and hospital admissions [8]. The impact of chronic diseases is worse in developing countries like Nigeria due to limitations in technological advancement for prompt diagnosis and treatment. [9]. The need for regular clinic visits could strain the patient's transportation cost and loss of income, leading to poor health outcomes. In addition, long waiting hours at the health facility could limit the adherence level of a patient. [10].

The scarce or inadequate healthcare funding and inefficient health system are reasons for Nigeria's chronic illness burden. In most developing nations, such as Nigeria, models, and techniques to adequately respond to the design of interventions to tackle the needs of communities and individuals are typically inadequate [11]. The emergence of a global pandemic (COVID-19)

prevented many chronically ill patients from visiting the hospital or booking appointments with doctors for fear of infection [12]. Amongst other things, depression, arising from a lack of attention to patients' mental health at the point of diagnosis and treatment, is one of the sufferings of chronic disease patients. [13]. To address the effects of a high chronic disease burden, there is a need for innovative delivery models in the healthcare system [14].

In Nigeria, medication dispensing for chronic diseases traditionally occurs within healthcare facilities, reflecting the historical development of the healthcare system. Patients typically obtain medications directly from hospitals or clinics during visits where healthcare professionals oversee the process. However, there's increasing recognition of the need for innovative approaches like smart lockers to improve accessibility and adherence in managing chronic diseases [15]

According to Mash and others [16], a model that involves patients and their families in selecting the most suitable methods of care is paramount to the delivery of healthcare to chronic disease patients, as it aims to promote fairness in healthcare delivery, improvement in quality of care, and patient safety, reduced healthcare expenditure for both patient and health care facilities, improve patient satisfaction, and enhance adherence to programs for clinical management [14].

In the health care context, smart lockers are automated machines that enable patients to access medications without delay rather than wait long hours in the queue at the health facility or pharmacy. The lockers can be placed at locations like shopping malls, retail stores, cinemas, etc., where users can easily access them [17]. Smart lockers and pharmaceutical dispensing devices are newer technologically driven processes that have been tested on a small scale. To decongest the clinics and reduce waiting time, Neo Hutiri developed the Pelebox smart locker, which allows patients access to the automated drug dispensing machine using an automated unique PIN-

generating model for patient use only. This one-time unique PIN allows patients to retrieve medication from the machine [10]. These automated machines have been tested in Johannesburg, South Africa. They provide faster and easier access to essential medicines for those with HIV and other chronic illnesses. The devices are linked to public health facilities that care for patients with chronic diseases, ensuring they get and follow through on appropriate treatments. Users of the machines are given a printed receipt that includes the date for their next appointment and an SMS reminder. If, for any reason, patients show up late to pick up their medications, the system notifies the pharmacists [18].

Nigerian health policy has evolved dramatically over the last 60 years. Yet, there is still a deficiency in the quality of the delivery in the healthcare system to improve the country's expectations. [18]. According to the World Bank's UHC Service Coverage Index, Nigeria ranks low in universal health coverage (UHC) [19]. Whether health services are delivered in a way that ensures optimal client and stakeholder satisfaction remains a significant concern for both public and private health institutions. [19].

Primary healthcare facilities struggle to meet the increasing patient demand, lacking sufficient infrastructure, human resources, and efficient record-keeping systems. This leads to long wait times for medication, causing patients to lose income due to missed workdays. Furthermore, facilities are often overwhelmed by stable patients seeking medication refills, leading to delays in treatment and poor adherence to chronic condition management. [20].

Smart lockers have remarkably reduced long waiting hours at health facilities, reduced the financial burden on patients and facilities, and increased adherence to medication among Persons Living with HIV (PLHIV) and chronic disease patients in South Africa. Introducing a similar

system in Nigeria will reduce the burden of health services provided to the already overwhelmed healthcare workforce and largely improve the healthcare system. This is because no previous studies have identified, documented, or evaluated using smart lockers in Nigeria for dispensing chronic disease medication. Therefore, this study aimed to describe patients' and healthcare workers' preferences for using smart lockers to dispense chronic disease medications in Nigeria [21].

Method

Study design

This study used a descriptive cross-sectional research design. The survey was part of a broader study assessing the feasibility and acceptability of smart lockers in dispensing chronic disease medication in Nigeria.

Study population

The populations for this study were:

Healthcare workers who provide care and treatment services to individuals with chronic diseases and persons living with one or more chronic diseases.

Inclusion criteria

1. People above the age of 18, residents in Nigeria who have a chronic disease that necessitates regular medication refills.
2. Healthcare workers for chronically ill individuals.

Sample size determination

Patients

According to Faronbi and co [9], the percentage of chronic diseases in Nigeria is 64.9%. This was used as a proxy to estimate, the population prevalence of chronic diseases, given that the population of Nigeria is 200 million by United Nations Population Fund in 2021. This was entered into the Raosoft® sample size calculator at a 4% margin of error, 95% confidence interval, and 50% expected response to yield a minimum sample size of 601.

Healthcare workers (HCWs)

According to Adebayor and co [22], the estimated number of healthcare workers (HCW) was 621, 205. This was used as a proxy for HCW to care for patients with chronic illnesses. This was imputed into the Raosoft® sample size calculator at a 5% margin of error, 95% confidence interval, and 50% expected response to yield a minimum sample size of 384.

Sampling

A stratified random sampling technique to identify patients with chronic illnesses within the sites where Antiretroviral Therapy (ART) programs are implemented in the country was used by two Implementing Partners (IPs). The participants were stratified based on gender, i.e., 50% male and 50% female, to ensure adequate coverage of the target population. The IPs or their representatives within each study site served as field supervisors for data collection during the study. Copies of the questionnaire were printed and distributed to all participating IPs or completed online through a questionnaire transcribed into REDCap.

Secondly, the survey was advertised online via social networks, including but not limited to private messaging, electronic mail, social network sites, and groups through platforms such as WhatsApp, Facebook, Instagram, and LinkedIn. Potential participants were privy to information about the study and screened based on the inclusion criteria for the study.

Data collection method

A semi-structured questionnaire was used. It contained questions designed to capture participants' socio-demographic characteristics and their responses to their preferences for accessibility, methods of engagement, operability, and eligible populations that would be most suited to use smart lockers for chronic medication dispensing. Potential participants who agreed to participate but did not have access to a mobile device or had poor internet connectivity were given a paper-based questionnaire to complete independently or with assistance from a survey team member. The completed forms were returned to the survey team and then carefully transcribed into the online survey to ensure better data management. The data entry was completed by a trained research assistant and verified by the research coordinator to ensure good data quality.

There was also a provision for participants willing and able to fill out the online survey directly via REDCap. The online survey used minimal data connectivity via multiple network options on the participant's device. A click on the hyperlink directed the participants to the entry page of the survey. A click on the submit button marked the end of the survey. There was no room for participants to edit their responses after submission. No personally identifiable data was collected from the participants. Informed consent was obtained from each participant by ticking the "I agree" checkbox on the entry page of the survey questionnaire before completing the questionnaire. Data collection was conducted between November – December 2021.

Data analysis

Frequency distributions were used to present the results of demographic data. Bivariate associations were used to estimate the degree of association between the HCWs and patients' preferences for using smart lockers in dispensing chronic disease medication. Chi-square test was

performed using a 95% CI, and statistical significance was established at $p<0.05$. Stata version 15 was used for analysis.

Ethical Statement

Informed consent

The entry page of the survey contained information on the study objectives, eligibility criteria, data privacy and researchers' disclaimers. Informed consent was obtained from all participants.

Confidentiality

All entries were recorded anonymously. Personally identifiable information was not collected from the participants. The privacy of the subjects' information was guaranteed.

Risks and benefits

There were no adverse effects that affected the rights and welfare of the subjects. There were no direct benefits associated with participation in the study.

Ethical clearance

The study protocol was submitted for review and approval to the Nigeria Health Research Ethics Committee (NHREC). Permission was also obtained from the administrators of the health facilities where data collection was conducted.

Results

1,180 survey entries were received, and 47 (4.0%) responses were excluded from data analysis because they did not meet the inclusion criteria. Of the 1,133 responses included in the analysis, 405 were received from HCWs, and 728 were from persons living with chronic illnesses in Nigeria.

Table 1. Demographics of the participants of patients with chronic diseases and health care workers in 5 states in Nigeria

Variables	HCWs, n = 405	Patients, n = 728
**Gender**		
Male	208 (51.4%)	321 (44.1%)
Female	197 (48.6%)	407 (55.9%)
**Age**		
18 – 35	275 (67.9%)	338 (46.4%)
36 – 60	125 (30.9%)	365 (50.1%)
> 60	5 (1.2%)	25 (3.4%)
**Marital Status**		
Single	198 (48.9%)	187 (25.7%)
Married	197 (48.6%)	419 (57.6%)
Separated	10 (2.5%)	122 (16.8%)
**Highest Educational Qualification**		
No formal Education	2 (0.5%)	97 (13.3%)
Primary Education	7 (1.7%)	135 (18.5%)
Secondary Education	44 (10.9%)	295 (40.5%)
Post-Secondary Education	352 (86.9%)	201 (27.6%)

Table 1 shows the demographic characteristics of the participants with chronic diseases and healthcare workers in 5 states in Nigeria. Most of the respondents in the patient survey were female (55.9%), whereas most HCWs were male (51.4%). 67.9% were between the age group of 18-35 years (HCWs), and 50.1 % of the participants were within the age group 36-60 years for patients, respectively. 48.9% of the HCWs were single, and 57.6% of the participants were married for Patients, respectively. Most participants had Post-Secondary Education (86.9%) and Secondary Education (40.5%) for HCWs and Patients groups, respectively.

Table 2. The most suitable Personnel to load the drugs into the Smart Lockers in 5 states in Nigeria.

Personnel	HCWs, n = 405
HIV Case Manager	57 (14.1%)
Monitoring and Evaluation (M&E staff)	6 (1.5%)

Nurse	7 (1.7%)
Pharmacist or other pharmacy staff	262 (64.7%)
Specific staff employed for this sole purpose	66 (16.3%)
Others	7 (1.7%)

Others: Trained personnel and staff

Among healthcare workers (HCW), Table 2 revealed that the most preferred personnel to load drugs into the smart lockers are pharmacists or other pharmacy staff (64.7%) against the Monitoring and evaluation (M&E) staff who had the lowest responses rate of (1.5%). Specific staff employed for this sole purpose was the next most preferred personnel to load drugs into smart lockers and HIV case managers had response rates of (16.3%) and (14.1%) respectively compared to Nurses and other professions who had lower response at (1.7%).

Table 3. Preferences for the use of smart lockers in dispensing chronic disease medications in 5 states in Nigeria.

Variables	HCWs, n = 405	Patients, n = 728	p - value
**How might patient orientation be conducted on the use of the smart lockers?**			
Through audio-visuals	93 (23.0%)	147 (20.2%)	$p = 0.25$
Through printed material	91 (22.5%)	146 (20.1%)	
One-on-One counseling by Healthcare Provider	218 (53.8%)	423 (58.1%)	
Others[1]	3 (0.7%)	12 (1.6%)	
**What would be the best location for placing the smart lockers for access to patients?**			
Bus stop	2 (0.5%)	17 (2.3%)	$p < 0.05$
Community pharmacy	102 (25.2%)	139 (19.1%)	
Hospital or Clinic	279 (68.9%)	521 (71.6%)	
Police station	5 (1.2%)	6 (0.8%)	
Religious institution	9 (2.2%)	34 (4.7%)	
Others[2]	8 (2.0%)	11 (1.5%)	
**How best might patients receive notification of drug dispensing?**			
Email	34 (8.4%)	47 (6.5%)	$p = 0.63$
Text Message	165 (40.7%)	314 (43.1%)	
Phone call	199 (49.1%)	355 (48.8%)	
Others[3]	7 (1.7%)	12 (1.6%)	

**What operating time would you recommend for patients to access the smart lockers?**			
Morning (6 am – 12 noon)	77 (19.0%)	166 (22.8%)	$p < 0.05$
Afternoon (12 noon – 4 pm)	43 (10.6%)	89 (12.2%)	
Evening (4 pm – 10 pm)	20 (4.9%)	90 (12.4%)	
24-hour access	265 (65.4%)	383 (52.6%)	
**What patients that might benefit from using the smart lockers to collect their medication**			
Patients with HIV	175 (43.2%)	334 (45.9%)	$p < 0.05$
Patients with Cancer	41 (10.1%)	71 (9.8%)	
Patients with Diabetes	49 (12.1%)	81 (11.1%)	
Patients with Hypertension	47 (11.6%)	95 (13.0%)	
Patients with Tuberculosis	90 (22.2%)	106 (14.6%)	
Others[4]	3 (0.7%)	41 (5.7%)	
**For which of these age groups can the smart lockers be used in dispensing medication?**			
1 – 12 years	1 (0.2%)	8 (1.1%)	$p < 0.05$
13 – 17 years	5 (1.2%)	14 (1.9%)	
18 – 40 years	328 (81.0%)	513 (70.5%)	
41 – 60 years	55 (13.6%)	128 (17.6%)	
Above 60 years	16 (4.0%)	24 (3.3%)	
Others[5]	0 (0.0%)	41 (5.6%)	

Others[1]: Unspecified.
Others[2]: Any convenient location, Bank, Post office.
Others[3]: Religious announcements, cluster collectors, disseminating information to other patients, face-to-face, home visits.
Others[4]: Asthmatic, Surgical cases, Malaria, Hepatitis with stroke
Others[5]: Unspecified

The preference for one-on-one counselling as the best method of orientating patients on using smart lockers for dispensing chronic disease medication was higher in both the HWCs (53.8%) and the patients (58.1%), which signifies a preference for the use of smart lockers as a method for dispensing chronic disease medication, and this was no significant difference between the HCWs and the patients ($p = 0.25$). Most participants preferred a hospital or clinic as the secure and easily accessible location for the smart lockers. The preference was higher amongst patients (71.6%) than HWCs (68.9%). This was statistically significant with ($p < 0.05$).

Regarding the method of notification for drug delivery, the majority of HCWs (49.1%) and patients (48.8%) preferred using a phone call to notify patients when their drugs were dispensed into the smart lockers. There was no significant difference between (HCW and patients ($p=0.63$).

There was a significant difference between the HCWs and Patients as both the HCWs (65.4%) and the patients (52.6%) accepted and preferred 24-hour access for the pick-up of drugs from the smart lockers with ($p<0.05$). Regarding the most suitable population to use the smart lockers, most participants responded that patients with HIV (HCWs – 43.2%, Patients – 45.9%) between the ages of 18 – 40 (HCWs – 81.0%, Patients – 70.5%) would benefit the most from using the smart lockers for dispensing their medication (Table 3).

Discussion

The study explored the potential benefits of implementing smart lockers for dispensing chronic disease medication in Nigeria. Specifically, it aimed to address the burden on healthcare services and the lack of previous research on this topic in Nigeria. According to our survey findings, smart lockers can be strategically placed close to a secure clinic or hospital, allowing patients in the surrounding area 24-hour access to pick up their prescription drugs. Medication can be administered by calling the patients. Patients, or end users, need to be trained on how to use the smart lockers; getting one-on-one counselling from their doctors is the best course of action. Our study explored the preferences for using smart lockers for dispensing chronic disease medication focusing on 5 states in Nigeria. According to Angell and others [23], the quality of healthcare systems in low-middle-income countries like Nigeria is poor. This contributes to the increase in the burden of illnesses and global health costs. There is an increasing focus on strengthening health systems by investing in improving the quality of health services and creating equitable access to high-quality, people-centred care in building trust in the health system. To achieve these goals, the perspectives, and preferences of patients and HCWs must be considered [24].

The overwhelming responses stemming from patients and HCWs regarding the populations best suited to use the smart lockers for chronic disease dispensing indicates that persons living with

HIV between 18 and 40 years are the most suitable populations to use the smart lockers. This is likely due to the prevalence of HIV in Nigeria, with the highest HIV prevalence rates recorded among women between the ages of 25 and 34 [25, 26]. This may also be because most respondents in the survey were between the ages of 18 and 40. Additionally, decentralized care, such as community-based drug delivery and ART refills from community pharmacies, is popular in managing diseases such as HIV and Tuberculosis in Nigeria, unlike other chronic diseases [27, 28]. This would indicate a lesser resistance to adopting smart lockers as another model for decentralized care. However, the incidence of other chronic diseases, such as diabetes, hypertension, and stroke, is higher in older populations aged 36 - 60 and above 60 years [29, 30]. There is increasing advocacy for improving person-centred care for older adults with chronic illnesses by transferring care from healthcare facilities to home and community-based models [31].

While most respondents advocate placing the lockers within a health institution such as a hospital, clinic, or community pharmacy, very few were open to using other locations such as bus stops, religious institutions, and police stations. This may be due to concerns relating to safety, privacy, and stigmatization [32]. Literature suggests that the rates of stigmatization differ based on the health condition, with persons suffering from HIV, Tuberculosis, and mental health conditions being among the most severely affected by stigmatization. These factors are often linked to a lack of awareness, beliefs, and institutionalized practices within healthcare facilities and communities [33, 34]. Decentralizing care and bringing the lockers to non-health-related institutions further expose patients to stigmatization and lack of privacy risks.

The patients and HCWs reported that they preferred smart lockers in a hospital or clinic. The sentiments behind this preference may be similar to those reported by Peng and others [35], who found that patients living with chronic diseases preferred to access care through primary healthcare

(PHC) services, provided that the distance to the service provider was short and that they had access to capable HCWs. The use of designated areas in the community close to the patients, such as religious institutions or retail pharmacies for drug pick-ups for chronic non-communicable diseases, is reported to limit the patient's need for physician consultation to issues such as an error in prescription, delay in delivery and adverse drug reactions [36].

Results of our study also indicated that one-on-one counseling would be the most effective method of orientating patients using smart lockers. This may be linked to factors such as the literacy level of patients and their comprehension of health-related information disseminated through mediums such as audio-visual tools and printed materials. Most patients in our study reported having completed a secondary school education (40.5%). Patient literacy has been suggested to influence individuals' health-seeking attitudes, impacting their ability to read, understand and act upon health-related information [37]. Additionally, many patients rely heavily on the opinions of their HCWs and are more likely to adopt treatment measures that a trusted physician suggests. This is especially applicable in the case of chronically ill persons who require ongoing care and have developed a strong relationship with their HCW over time [38]. Having one-on-one contact with a Healthcare provider may overcome the barriers to understanding information about using smart lockers and increase their likelihood of adopting smart lockers for collecting their medication. A study by Donelan and others [39] indicates that healthcare services should be made available to patients at any time of the day, considering the technological advancement the world is experiencing. Telemedicine affords a patient access to the physician without time restrictions and not necessarily having to go into the hospital [40]. This points to the agreement found in this study from HCW and patients' responses to access time to smart lockers. Most patients and HCWs have

the same line of thought that smart lockers for drug dispensing should be placed at the hospitals or clinics and made operational for 24 hours for easy access.

Furthermore, the usage of questionnaires may have resulted in self-selection bias, as respondents may have an essentially distinctive characteristic that affected their decision to prefer or not to prefer the use of smart lockers.

Due to the characteristics of the hospitals chosen and the fact that the HIV program provides the most extensive organized chronic care programs in those locations, the study has some limitations that may limit the generalizability of the findings to other chronic diseases.

In our study, most respondents opined that a phone call would be the best method for notifying patients when their drugs are dispensed. The use of smartphones has been reported to enhance patient drug adherence. Short Message Service (SMS) and synchronized data transmission applications enable healthcare providers to send reminders and keep up with patients' drug adherence [41]. However, the ownership of mobile phones and smartphones in Nigeria is limited. Limited network access in certain areas may also hinder the quick and seamless transmission of electronic mail and SMS [42]. This may be the reason why most of the respondents prefer receiving a phone call notification when their drugs are dispensed. This would be easier to track and ensure that the patients are adequately informed when their prescriptions are dispensed.

Conclusion

This is probably the first study assessing the preferences for using smart lockers in dispensing chronic disease medication among HCWs and persons living with chronic illnesses in Nigeria. The findings suggest that smart lockers may best serve PLHIV between the ages of 18 and 40 and this technology (Smart lockers) may be placed within a hospital or clinic that is within reach of patients

in the community. In terms of engagement and operationality, enabling 24-hour access to the smart locker for drug pick-up and using phone calls to notify patients when their drugs are dispensed would be best suited for dispensing patient medication and the best method for orientating patients on the use of smart lockers may be one-on-one counselling from their healthcare providers. Additional research may be conducted to develop models for using smart lockers for dispensing chronic disease medications to other populations of chronically ill persons in Nigeria.

Acknowledgement

We would like to thank all those who contributed in one way or another to the collection and analysis of this data.

Funding:

The study was funded by Georgetown University Medical Center (GUMC), Dean of Research. However, the funder did not play any role in the research.

S1 Table: Demographics of the Participants of Patients with Chronic Diseases and Health Care Workers in 5 States in Nigeria

S2 Table: The most suitable personnel to load the drugs into the smart lockers in 5 states in Nigeria.

S3 Table: Preferences for the use of smart lockers in dispensing chronic disease medications in 5 states in Nigeria.

S1 Appendix: Distribution of states and facilities

S2 Appendix: Supporting data.

References

1. CDC (2021). National Center for Chronic Disease Prevention and Health Promotion. About Chronic Diseases. Retrieved 25 March 2022 from https://www.cdc.gov/chronicdisease/about/index.htm.
2. Anderson, E., & Durstine, J. L. (2019). Physical activity, exercise, and chronic diseases: A brief review. *Sports Medicine and Health Science*, *1*(1), 3-10.
3. Kunnumakkara, A. B., Sailo, B. L., Banik, K., Harsha, C., Prasad, S., Gupta, S. C., et al. (2018). Chronic diseases, inflammation, and spices: how are they linked? *Journal of translational medicine*, *16*(1), 14. https://doi.org/10.1186/s12967-018-1381-2.
4. World Health Organization (2020). WHO reveals leading causes of death and disability worldwide: 2000-2019. Retrieved 25 March 2020 from https://www.who.int/news/item/09-12-2020-who-reveals-leading-causes-of-death-and-disability-worldwide-2000-2019.
5. World Health Organization (2018). Noncommunicable diseases country profiles 2018. Retrieved 25 March 2022 from https://apps.who.int/iris/handle/10665/274512.
6. World Health Organization (2018).Global Health Observatory Data; Premature NCD deaths.Available from: https://www.who.int/gho/ncd/mortality_morbidity/ncd_premature_text/en/.
7. Sav, A., Salehi, A., Mair, F.S. *et al.* Measuring the burden of treatment for chronic disease: implications of a scoping review of the literature. *BMC Med Res Methodol* 17, 140 (2017). https://doi.org/10.1186/s12874-017-0411-8.
8. Hajat, C., & Stein, E. (2018). The global burden of multiple chronic conditions: A narrative review. *Preventive medicine reports, 12,* 284–293. https://doi.org/10.1016/j.pmedr.2018.10.008

9. Faronbi, J. O., Ademuyiwa, I. Y., & Olaogun, A. A. (2020). Patterns of chronic illness among older patients attending a university hospital in Nigeria. *Ghana medical journal*, *54*(1), 42–47. https://doi.org/10.4314/gmj.v54i1.7.
10. De Villiers, K. Bridging the health inequality gap: an examination of South Africa's social innovation in health landscape. *Infect Dis Poverty* 10, 19 (2021). https://doi.org/10.1186/s40249-021-00804-9.
11. Idris, I.O., Oguntade, A.S., Mensah, E.A. *et al.* Prevalence of non-communicable diseases and its risk factors among Ijegun-Isheri Osun residents in Lagos State, Nigeria: a community based cross-sectional study. *BMC Public Health* 20, 1258 (2020). https://doi.org/10.1186/s12889-020-09349-2.
12. Büssing, A., Hübner, J., Walter, S., Gießler, W., & Büntzel, J. (2020). Tumor patients perceived changes of specific attitudes, perceptions, and behaviors due to the COVID-19 pandemic and its relation to reduced wellbeing. *Frontiers in psychiatry*, 883.
13. Jiang, Ch., Zhu, F. & Qin, (2020). Tt. Relationships between Chronic Diseases and Depression among Middle-aged and Elderly People in China: A Prospective Study from CHARLS. *CURR MED SCI* 40, 858–870 (2020). https://doi.org/10.1007/s11596-020-2270-5.
14. Yakubu, K., Malan, Z., Colon-Gonzalez, M. C., & Mash, B. (2018). Perceptions about family-centred care among adult patients with chronic diseases at a general outpatient clinic in Nigeria. *African journal of primary health care & family medicine*, *10*(1), e1–e11. https://doi.org/10.4102/phcfm.v10i1.1739.
15. Yusuff KB, Abd' Wassi Sanni. Itinerant vending of medicines inside buses in Nigeria: vending strategies, dominant themes and medicine-related information provided. PubMed. 2011 Jul 1;

16. Mash, R., Christian, C., & Chigwanda, R. V. (2021). Alternative mechanisms for delivery of medication in South Africa: A scoping review. *South African family practice: official journal of the South African Academy of Family Practice/Primary Care*, *63*(1), e1–e8. https://doi.org/10.4102/safp.v63i1.5274.

17. Kiousis, V., Nathanail, E., Karakikes, I. (2019). Assessing Traffic and Environmental Impacts of Smart Lockers Logistics Measure in a Medium-Sized Municipality of Athens. In: Nathanail, E., Karakikes, I. (eds) Data Analytics: Paving the Way to Sustainable Urban Mobility. CSUM 2018. Advances in Intelligent Systems and Computing, vol 879. Springer, Cham. https://doi.org/10.1007/978-3-030-02305-8_74.

18. UNAIDS (2018). South Africa tests ATMs for medicine. Retrieved 28 March 2022 fromhttps://www.unaids.org/en/resources/presscentre/featurestories/2018/may/south-africa-tests-atms-for-medicine.

19. Asakitikpi, A. E. (2019). Chapter: Healthcare Coverage and Affordability in Nigeria: An Alternative Model to Equitable Healthcare Delivery. DOI: 10.5772/intechopen.85978

20. McKing, I. Ifunaya, C. (2021) Improving access, quality, and efficiency in health care delivery in Nigeria: a perspective. PAMJ - One Health. 2021; 5:3. doi: 10.11604/pamj-oh.2021.5.3.28204.

21. Ephraim-Emmanuel B, Adigwe A, Oyeghe R, Ogaji D (2018) 2875. Quality of health care in Nigeria: a myth or a reality. *International Journal of Research in Medical Science*- DOI - 10.18203/2320-6012.ijrms20183621.

22. Adebayo, O., Labiran, A., Emerenini, F. C. and Omoruyi, L. (2016). Health Workforce for 2016-2030: Will Nigeria have enough? International Journal of Innovative Healthcare Research. 4(1), 9-16.

23. Angell, B., Sanuade, O., Adetifa, I.M.O., Okeke, I.N., Adamu, A.L., Aliyu, M.H., et al. (2022). Population health outcomes in Nigeria compared with other west African countries, 1998–2019: a systematic analysis for the Global Burden of Disease Study. The Lancet, [online] 399(10330), pp.1117–1129. doi:https://doi.org/10.1016/S0140-6736(21)02722-7.
24. Institute for Health Metrics and Evaluation (IHME) (2020). GBD Compare Data Visualization. Seattle, WA: IHME, University of Washington, 2020. Retrieved 6 April 2022 from http://vizhub.healthdata.org/gbd-compare.
25. Federal Ministry of Health, Nigeria. Nigeria HIV/AIDS Indicator and Impact Survey (NAIIS) 2018: Technical Report. Abuja, Nigeria. October 2019. Accessed 11 April 2022 from http://ciheb.org/media/SOM/Microsites/CIHEB/documents/NAIIS-Report-2018.pdf
26. Rabkin, M., Strauss, M., Mantell, J. E., Mapingure, M., Masvawure, T. B., Lamb, M. R., et al. (2020). Optimizing differentiated treatment models for people living with HIV in urban Zimbabwe: Findings from a mixed methods study. *PloS one*, *15*(1), e0228148. https://doi.org/10.1371/journal.pone.0228148
27. Magadzire, B.P., Marchal, B. & Ward, K. (2016). Novel models to improve access to medicines for chronic diseases in South Africa: an analysis of stakeholder perspectives on community-based distribution models. *Journal of Pharmaceutical Policy and Practice,* **9,** 28 https://doi.org/10.1186/s40545-016-0082-6
28. Muthelo, L., Nemagumoni, T., Mothiba, T.M., Phukubje, A.T. and Mabila, L.N. (2020). Experiences of Professional Nurses Regarding the Implementation of a Central Chronic Medicine Dispensing and Distribution Program at Primary Health Care Facilities in South Africa. The Open Public Health Journal, 13(1), pp.477–483. doi:https://doi.org/10.2174/1874944502013010477.

29. Zhao, J., Yan, C., Han, D., Wu, Y., Liao, H., Ma, Y., et al. (2022). Inequity in the healthcare utilization among latent classes of elderly people with chronic diseases and decomposition analysis in China. BMC Geriatrics, 22(1). doi:https://doi.org/10.1186/s12877-022-03538-x.

30. Nolte, E., Merkur, S., Anders Anell and European Observatory On Health Systems And Policies (2020). Achieving person-centred health systems : evidence, strategies and challenges. Cambridge ; New York, Ny: Cambridge University Press.

31. Dong R, Leung C, Naert MN, Naanyu V, Kiptoo P, Matelong W, et al. (2021) Chronic disease stigma, skepticism of the health system, and socio-economic fragility: Qualitative assessment of factors impacting receptiveness to group medical visits and microfinance for non-communicable disease care in rural Kenya. *PLoS ONE* 16(6): e0248496. https://doi.org/10.1371/journal.pone.0248496

32. Idemudia, E. S., Olasupo, M. O., & Modibo, M. W. (2018). Stigma and chronic illness: A comparative study of people living with HIV and/or AIDS and people living with hypertension in Limpopo Province, South Africa. *Curationis*, *41*(1), e1–e5. https://doi.org/10.4102/curationis.v41i1.1879

33. Nyblade, L., Stockton, M.A., Giger, K., Bond, V., Ekstrand, M. L., Mc Lean, R., et al. (2019). Stigma in health facilities: why it matters and how we can change it. *BMC Med* **17,** 25. https://doi.org/10.1186/s12916-019-1256-2

34. Peng Y, Jiang M, Shen X, Li X, Jia E, Xiong J. (2020). Preferences for Primary Healthcare Services Among Older Adults with Chronic Disease: A Discrete Choice Experiment. Patient Prefer Adherence;14:1625-1637. Doi: https://doi.org/10.2147/PPA.S265093

35. Krzyzaniak, N., Bajorek, B. A global perspective of the roles of the pharmacist in the NICU (2017). *International Journal of Pharmacy Practice*, Volume 25, Issue 2, April 2017, Pages 107–120, https://doi.org/10.1111/ijpp.12284

36. Grilo, A. M. , Santos, M. C. d. , Isabel Gomes, A., & Rita, J. S. (2017). Promoting Patient-Centered Care in Chronic Disease. In (Ed.), Patient Centered Medicine. IntechOpen. https://doi.org/10.5772/67380

37. Coventry, P. A., Fisher, L., Kenning, C., Bee, P., & Bower, P. (2014). Capacity, responsibility, and motivation: a critical qualitative evaluation of patient and practitioner views about barriers to self-management in people with multimorbidity. *BMC health services research*, *14*, 536. https://doi.org/10.1186/s12913-014-0536-y

38. Pritchard, D. E., Moeckel, F., Villa, M. S., Housman, L. T., McCarty, C. A., and McLeod, H. L. (2017). Strategies for integrating personalized medicine into healthcare practice. *Personalized Medicine*, 14,2, 141-152. Doi: 10.2217/PME-2016-0064

39. Donelan, K., Barreto, E. A., Sossong, S., Michael, C., Estrada, J. J., Cohen, A. B., et al. (2019). Patient and clinician experiences with telehealth for patient follow-up care. *The American journal of managed care*, *25*(1), 40–44.

40. Reed, M. E., Huang, J., Graetz, I., Lee, C., Muelly, E., Kennedy, C., et al. (2020). Patient Characteristics Associated With Choosing a Telemedicine Visit vs Office Visit With the Same Primary Care Clinicians. *JAMA network open*, *3*(6), e205873. https://doi.org/10.1001/jamanetworkopen.2020.5873

41. Al-Hassany L, Kloosterboer SM, Dierckx B, Koch BCP (2019). Assessing methods of measuring medication adherence in chronically ill children–a narrative review. *Patient Preference and Adherence*; 13:1175-1189. Doi: https://doi.org/10.2147/PPA.S200058

42. Ivan Forenbacher, Siniša Husnjak, Ivan Cvitić, Ivan Jovović, (2019). Determinants of mobile phone ownership in Nigeria. *Telecommunications Policy*,43, 7, 101812. Doi: https://doi.org/10.1016/j.telpol.2019.03.00

Click here to access/download
Supporting Information
Manuscript 3_Supporting Data_1_11_2023.xlsx

Click here to access/download
Supporting Information
STROBE Checklist_SLS_Man 3_31_10_2023.docx

Click here to access/download
Supporting Information
PLOS ONE Clinical Studies Checklist_Man
3_31_10_2023.docx

Click here to access/download
Supporting Information
Study protocol- SLS.pdf

Click here to access/download
Supporting Information
NHREC Approval.pdf

Click here to access/download
Supporting Information
Approval of Protocol from GU_04Jan2022.pdf

Click here to access/download
Supporting Information
S1 Appendix. Distribution of States and Facilities.docx

Click here to access/download
Supporting Information
Inclusivity-in-global-research-questionnaire 13_2_2024.docx

~~Patients and Healthcare Workers' Preferences for Using Smart Lockers in Dispensing Chronic Disease Medication in Nigeria.~~

Patients and Healthcare Workers' Preferences for Using Smart Lockers in Accessing and Dispensing Chronic Disease Medication in Nigeria: Findings from a Descriptive Cross-Sectional Study.

Formatted: Font:

Ibrahim Bola Gobir[1*], Samson Agboola[2], Havilah Onyinyechi Nnadozie[2], Helen Adamu[2], Fatimah Ohunene Sanni[2], Aisha Adamu[2], Azeez Akanbi Bello ~~Akanbi~~[2], Angmun Suzzy Otubo[3], Piring'ar Mercy Niyang[1]

[1]Center for Global Health Practice and Impact, Georgetown University, Washington DC, United States of America

[2]Georgetown Global Health Nigeria, Federal Capital Territory, Abuja, Nigeria

[3]Savannah Health System Innovation Limited, Federal Capital Territory, Abuja, Nigeria

[*]Corresponding author

Email: ibg7@georgetown.edu (IBG)

Abstract

The use of smart locker technology has been beneficial for patients with chronic diseases who require regular medication and face challenges accessing healthcare facilities due to distance, time, or mobility issues. ~~Objectives:~~ The objective of this study was to describe patients’ ~~-~~and healthcare workers’ preferences for using smart lockers for dispensing chronic disease medications in Nigeria. ~~Methods:~~ We employed a descriptive cross-sectional research design during our study. We focused on healthcare workers and patients with chronic diseases in 5~~6~~ Nigerian states of Adamawa, Akwa Ibom, Cross River, Benue, and Niger between November and December 2021. Using a stratified random sampling technique, a semi-structured questionnaire was used to collect data from healthcare workers who provide care and treatment services to individuals with chronic diseases and persons living with one or more chronic diseases and/or their clients (participants). ~~Results:~~ A total of 1,133 participants (HCWs – 405 and Patients – 728) were included in our study. The total number of responses received during the study was 1,180, and 47 (4.0%) were excluded from data analysis because they did not meet the inclusion criteria.~~-~~ Our findings reflect that the suitable populations to use smart lockers were HCWs and patients with HIV (HCWs – n =175, 43.2%, Patients – n=334, 45.9%) between the ages of 18 and 40 years (HCWs – 81.0%, Patients – 70.5%).

~~HCWs and patients with HIV (HCWs – 43.2%, Patients – 45.9%) between the ages of 18 and 40 years (HCWs – 81.0%, Patients – 70.5%) are the most suitable population to use the smart lockers.~~

~~Out of the 180 responses received for the study, 47 (4.0%) were excluded from data analysis because they did not meet the inclusion criteria.~~ There were no significant differences between the HCWs and the patients’ choices on the method of orientation, i.e., they both preferred (one-

Formatted: Font: 12 pt

on-one counselling with their healthcare providers) (p = *0.25*) and notification for drug pick up (phone call) (p = *0.63*). The preference for smart lockers location and the accessibility time between HCWs and patients were statistically significant ($p < 0.05$); the hospital or clinic as a preferred location was higher amongst patients (71.6%) than HCWs (68.9%) and the 24-hour access to smart lockers preference was lower amongst the patients (52.6%) than the HCWs (65.4%). ~~**Conclusion:**~~ The study findings revealed that the preferred age group that smart lockers may best serve was Persons Living with HIV (PLHIV) between 18 and 40 years old. The lockers may be placed within a secured hospital or clinic and within reach of patients in the community with 24-hour access to the smart locker for drug pick-up. The patients can be reached for medication dispensing through phone calls. The preferred method of orientation by both patients and HCWs on the use of smart lockers for dispensing medication is one-on-one counselling with their healthcare providers. ~~The end-users (patients) need orientation on using the smart lockers; the preferred method is one-on-one counselling from their healthcare providers.~~ ~~**Recommendation(s):**~~ Given that smart lockers have the potential to enhance medication adherence and improve health outcomes. The development of models for using smart lockers to dispense chronic disease medications to chronically ill persons in Nigeria and other populations is recommended.

Keywords: Smart lockers, Chronic diseases, Healthcare workers, Medication, Persons Living with HIV(PLHIV)

Background

Chronic disease is a sickness that lasts a long time, advances slowly, is caused by genetics, the environment, or poor lifestyle choices, and requires continuous medical care [1]. Chronic diseases were responsible for more than 28 million deaths worldwide in 1990. By 2008, this number had climbed to 36 million~~,~~ and 39 million in 2016 [2]. Chronic diseases remain the leading cause of death globally [3,4]. In Nigeria alone, chronic respiratory diseases, diabetes, cancer, and cardiovascular diseases were the cause of up to 29% of all deaths in 2018. [5]. The World Health Organization (WHO) reported a 20% probability of dying prematurely from non-communicable diseases (NCDs) in Nigeria [6]. ~~According to Adesina and others [6], Nigerians have a 20% chance of dying untimely from chronic diseases.~~ Evidence suggests that chronic disease patients experience multimorbidity because chronic diseases rarely occur in isolation. The burden of managing the treatment of chronic illnesses includes changes in lifestyle, behaviours, regular visits to the healthcare professional or clinic, and managing medications [7]. Multimorbidity is linked to significant increases in healthcare expenses and resources due to increased primary care and specialist physician services, prescription use, emergency room visits, and hospital admissions [8]. The impact of chronic diseases is worse in developing countries like Nigeria due to limitations in

technological advancement for prompt diagnosis and treatment. [9]. The need for regular clinic visits could strain the patient's transportation cost and loss of income, leading to poor health outcomes. In addition, long waiting hours at the health facility could limit the adherence level of a patient. [10].

The scarce or inadequate healthcare funding and inefficient health system are reasons for Nigeria's chronic illness burden. In most developing nations, such as Nigeria, models and techniques to adequately respond to the design of interventions to tackle the needs of communities and individuals are typically inadequate [11]. The emergence of a global pandemic (COVID-19) prevented many chronically ill patients from visiting the hospital or booking appointments with doctors for fear of infection [12]. Amongst other things, depression, arising from a lack of attention to patients' mental health at the point of diagnosis and treatment, is one of the sufferings of chronic disease patients. [13]. To address the effects of a high chronic disease burden, there is a need for innovative delivery models in the healthcare system [14].

In Nigeria, medication dispensing for chronic diseases traditionally occurs within healthcare facilities, reflecting the historical development of the healthcare system. Patients typically obtain medications directly from hospitals or clinics during visits where healthcare professionals oversee the process. However, there's increasing recognition of the need for innovative approaches like smart lockers to improve accessibility and adherence in managing chronic diseases [15]

According to Mash and others [~~15~~16], a model that involves patients and their families in selecting the most suitable methods of care is paramount to the delivery of healthcare to chronic disease patients, as it aims to promote fairness in healthcare delivery, improvement in quality of care, and

patient safety, reduced healthcare expenditure for both patient and health care facilities, improve patient satisfaction, and enhance adherence to programs for clinical management [14].

Formatted: No underline, Font color: Auto

A smart locker is an automated delivery machine used to deliver goods and has been reported to lessen the burden of home delivery services. The effect of using smart lockers includes a drop in traffic density, an increase in cargo utilization through shipment consolidation, and a reduction in the time it takes to handle products. [16]. Smart lockers have been designed to address the inefficiencies of repeated distribution to increase logistics efficiency [17]. Using smart lockers is a revolutionary logistics approach to reduce challenges caused by last-mile parcel deliveries while promoting sustainable urban mobility concepts. [18].

In the health care context, smart lockers are automated machines that enable patients to access medications without delay rather than wait long hours in the queue at the health facility or pharmacy. The lockers can be placed at locations like shopping malls, retail stores, cinemas, etc., where users can easily access them [1517]. Smart lockers and pharmaceutical dispensing devices are newer technologically driven processes that have been tested on a small scale. To decongest the clinics and reduce waiting time, Neo Hutiri developed the Pelebox smart locker, which allows patients access to the automated drug dispensing machine using an automated unique PIN-generating model for patient use only. This one-time unique PIN allows patients to retrieve medication from the machine [10]. These automated machines have been tested in Johannesburg, South Africa. They provide faster and easier access to essential medicines for those with HIV and other chronic illnesses. The devices are linked to public health facilities that care for patients with chronic diseases, ensuring they get and follow through on appropriate treatments. Users of the machines are given a printed receipt that includes the date for their next appointment and an SMS

reminder. If, for any reason, patients show up late to pick up their medications, the system notifies the pharmacists [1918].

Problem Statement

Nigerian health policy has evolved dramatically over the last 60 years. Yet, there is still a deficiency in the quality of the delivery in the healthcare system to improve the country's expectations. [1820]. According to the World Bank's UHC Service Coverage Index, Nigeria ranks low in universal health coverage (UHC) [1921]. Whether health services are delivered in a way that ensures optimal client and stakeholder satisfaction remains a significant concern for both public and private health institutions. [1922].

Primary healthcare facilities fail to keep up with rising patient numbers, and their infrastructure, human resources, supply chains, and traditional paper-based record-keeping models are generally inadequate. Patients face long waits for medication and are likely to be out of work for the day, consequently losing income. Institutions are frequently overburdened with stable patients who merely require medication refills. Patients may delay collecting their medications due to this unpleasant experience, resulting in poor adherence and treatment of chronic conditions. Primary healthcare facilities struggle to meet the increasing demand from patientspatient demand, lacking sufficient infrastructure, human resources, and efficient record-keeping systems. This leads to long wait times for medication, causing patients to lose income due to missed workdays. Furthermore, facilities are often overwhelmed by stable patients seeking medication refills, leading to delays in treatment and poor adherence to chronic condition management. [1520].

~~Justification~~

Smart lockers have remarkably reduced long waiting hours at health facilities, reduced the financial burden on patients and facilities, and increased adherence to medication among Persons Living with HIV (PLHIV) and chronic disease patients in South Africa. Introducing a similar system in Nigeria will reduce the burden of health services provided to the already overwhelmed healthcare workforce and largely improve the healthcare system. ~~A key objective of the study was to describe the preferences of patients and healthcare workers in Nigeria for using smart lockers to dispense chronic illness medications.~~

This is because ~~N~~no previous studies have identified, documented, or evaluated using smart lockers in Nigeria for dispensing chronic disease medication. Therefore, ~~this study sought to describe patients' and healthcare providers' preferences for using smart lockers in dispensing chronic disease medication. We hypothesized a significant relationship exists between patients' and healthcare workers' (HCWs) preferences for using smart lockers to dispense chronic disease medication in Nigeria.~~

~~Aim and Objectives~~

~~T~~the study aimed to describe patients' and healthcare workers' preferences for using smart lockers ~~for dispensing~~to dispense chronic disease medications in Nigeria.[21] ~~with the following specific objectives:~~

~~1. To describe the patients' and healthcare workers' preferences for using smart lockers in dispensing chronic disease medications in Nigeria.~~

Formatted: Space Before: 12 pt, Add space between paragraphs of the same style, No bullets or numbering

Method

Study design

This study used a descriptive cross-sectional research design. The survey was part of a broader study assessing the feasibility and acceptability of smart lockers in dispensing chronic disease medication in Nigeria.

Study population

The populations for this study were:

1. Healthcare workers who provide care and treatment services to individuals with chronic diseases.

2. Persons living with one or more chronic diseases.

Formatted: Space After: 8 pt

Inclusion criteria

1. People above the age of 18, residents in Nigeria who have a chronic disease that necessitates regular medication refills.
2. Healthcare workers for chronically ill individuals.

Sample size determination

Patients

According to Faronbi and co [9], the percentage of chronic diseases in Nigeria is 64.9%. This was used as a proxy to estimate the population prevalence of chronic diseases, given that the

population of Nigeria is 200 million by United Nations Population Fund in 2021. This was entered into ~~Raosoft® sample size calculator at 4% margin of error, 95% confidence interval~~the Raosoft® sample size calculator at a 4% margin of error, 95% confidence interval, and 50% expected response to yield a minimum sample size of 601.~~The estimated sample size for the patients is 601 and was determined using a 5% margin of error, 95% confidence level, 80% response distribution, and population size proposed by [9] based on the chronic disease prevalence in Nigeria.~~

Field Code Changed

Healthcare workers (HCWs)

According to Adebayor and co [22], the estimated number of healthcare workers (HCW) was 621~~,~~205. This was used as a proxy ~~to~~for HCW to ~~provide care to~~care for patients with chronic illnesses. This was imputed into ~~Raosoft® sample size calculator at 5% margin of error, 95% confidence interval~~the Raosoft® sample size calculator at a 5% margin of error, 95% confidence interval, and 50% expected response to yield a minimum sample size of 384. ~~The estimated sample size for the healthcare workers was 384. It was estimated using a 5% margin of error, 95% confidence level, 80% response distribution, and population size of healthcare workers in Nigeria, as proposed by [23].~~

Field Code Changed

Sampling

A stratified random sampling technique to identify patients with chronic illnesses within the sites where Antiretroviral Therapy (ART) programs are implemented in the country was used by two Implementing Partners (IPs). The participants were stratified based on gender, i.e., 50% male and

50% female, to ensure adequate coverage of the target population. The IPs or their representatives within each study site served as field supervisors for data collection during the study. Copies of the questionnaire were printed and distributed to all participating IPs or completed online through a questionnaire transcribed into REDCap.

Secondly, the survey was advertised online via social networks, including but not limited to private messaging, electronic mail, social network sites, and groups through platforms such as WhatsApp, Facebook, Instagram, and LinkedIn. Potential participants were privy to information about the study and screened based on the inclusion criteria for the study.

Data collection method

A semi-structured questionnaire was used. It contained questions designed to capture participants' socio-demographic characteristics and their responses ~~on their preferences for the~~ to their preferences for accessibility, methods of engagement, operability, and eligible populations that would be most suited to use smart lockers for chronic medication dispensing. Potential participants who agreed to participate but did not have access to a mobile device or had poor internet connectivity were given a paper-based questionnaire to complete independently or with assistance from a survey team member. The completed forms were returned to the survey team and then carefully transcribed into the online survey to ensure better data management. The data entry was completed by a trained research assistant and verified by the research coordinator to ensure good data quality.

There was also a provision for participants willing and able to fill out the online survey directly via REDCap. The online survey used minimal data connectivity via multiple network options on the participant's device. A click on the hyperlink directed the participants to the entry page of the

survey. A click on the submit button marked the end of the survey. There was no room for participants to edit their responses after submission. No personally identifiable data was collected from the participants. Informed consent was obtained from each participant by ticking the “I agree” checkbox on the entry page of the survey questionnaire before completing the questionnaire. Data collection was conducted between November – December 2021.

Data analysis

Frequency distributions were used to present the results of demographic data. Bivariate associations were used to estimate the degree of association between the HCWs and patients’ preferences for using ~~and locating~~ smart lockers in dispensing chronic disease medication. Chi-square test was performed using a 95% CI, and statistical significance was established at $p<0.05$. ~~STATA~~ Stata version 15 was used for analysis.

Ethical Statement

Informed consent

The entry page of the survey contained information on the study objectives, eligibility criteria, data privacy and researchers’ disclaimers. Informed consent was obtained from all participants. ~~If a participant ticks the “I agree” checkbox on the survey, it was considered sufficient to provide informed consent. Entries from participants who do not meet the inclusion criteria was not processed for data analysis.~~

Confidentiality

All entries were recorded anonymously. Personally identifiable information was not collected from the participants. Privacy of the subjects’ information was guaranteed.

Risks and benefits

There were no adverse effects that affected the right and welfare of the subjects. There were no direct benefits associated with participation in the study.

Ethical clearance

The study protocol was submitted for review and approval to the Nigeria Health Research Ethics Committee (NHREC). Permission was also obtained from the administrators of the health facilities where data collection was conducted..

Results

1,180 survey entries were received, and 47 (4.0%) responses were excluded from data analysis because they did not meet the inclusion criteria. Of the 1,133 responses included in the analysis, 405 were received from HCWs, and 728 were from persons living with chronic illnesses in Nigeria.

Table 1. Demographics of the Pparticipants of patients with chronic diseases and health care workers in 5 states in Nigeria

Variables	HCWs, n = 405	Patients, n = 728
**Gender**		
Male	208 (51.4%)	321 (44.1%)
Female	197 (48.6%)	407 (55.9%)
**Age**		
18 – 35	275 (67.9%)	338 (46.4%)
36 – 60	125 (30.9%)	365 (50.1%)
> 60	5 (1.2%)	25 (3.4%)
**Marital Status**		
Single	198 (48.9%)	187 (25.7%)
Married	197 (48.6%)	419 (57.6%)
Separated	10 (2.5%)	122 (16.8%)
**Highest Educational Qualification**		
No formal Education	2 (0.5%)	97 (13.3%)
Primary Education	7 (1.7%)	135 (18.5%)
Secondary Education	44 (10.9%)	295 (40.5%)
Post-Secondary Education	352 (86.9%)	201 (27.6%)

Table 1 shows the demographic characteristics of the participants . with chronic diseases and health care workers in 5 states in Nigeria. Most of the respondents in the patient survey were female (55.9%), whereas most HCWs were male (51.4%). 67.9% were between the age group of 18-35 years (HCWs), and 50.1 % of the participants were within the age group 36-60 years for patients, respectively. 48.9% of the HCWs were single, and 57.6% of the participants were married for Patients, respectively. Most participants had Post-Secondary Education (86.9%) and Secondary Education (40.5%) for HCWs and Patients groups, respectively.

~~Table 2. Participants' preferences for the use of smart lockers in dispensing chronic disease medications in Nigeria~~

~~Variables~~	~~HCWs, n = 405~~	~~Patients, n = 728~~
**~~The patients that might benefit from using the smart lockers to collect their medication~~**		
~~Patients with HIV~~	~~175 (43.2%)~~	~~334 (45.9%)~~
~~Patients with Cancer~~	~~41 (10.1%)~~	~~71 (9.8%)~~
~~Patients with Diabetes~~	~~49 (12.1%)~~	~~81 (11.1%)~~
~~Patients with Hypertension~~	~~47 (11.6%)~~	~~95 (13.0%)~~
~~Patients with Tuberculosis~~	~~90 (22.2%)~~	~~106 (14.6%)~~
~~Others[1]~~	~~3 (0.7%)~~	~~41 (5.7%)~~
**~~For which of these age groups can the smart lockers be used in dispensing medication?~~**		
~~1 – 12 years~~	~~1 (0.2%)~~	~~8 (1.1%)~~
~~13 – 17 years~~	~~5 (1.2%)~~	~~14 (1.9%)~~
~~18 – 40 years~~	~~328 (81.0%)~~	~~513 (70.5%)~~
~~41 – 60 years~~	~~55 (13.6%)~~	~~128 (17.6%)~~
~~Above 60 years~~	~~16 (4.0%)~~	~~24 (3.3%)~~
~~Others[2]~~		~~41 (5.6%)~~
**~~How might patient orientation be conducted on the use of the smart lockers?~~**		
~~Through audio-visuals~~	~~93 (23.0%)~~	~~147 (20.2%)~~
~~Through printed material~~	~~91 (22.5%)~~	~~146 (20.1%)~~
~~One-on-One counseling by Healthcare Provider~~	~~218 (53.8%)~~	~~423 (58.1%)~~
~~Others[2]~~	~~3 (0.7%)~~	~~12 (1.6%)~~
**~~What would be the best location for placing the smart lockers for access to patients?~~**		

Bus stop	2 (0.5%)	17 (2.3%)
Community pharmacy	102 (25.2%)	139 (19.1%)
Hospital or Clinic	279 (68.9%)	521 (71.6%)
Police station	5 (1.2%)	6 (0.8%)
Religious institution	9 (2.2%)	34 (4.7%)
Others[4]	8 (2.0%)	11 (1.5%)
**How best might patients receive notification of drug dispensing?**		
Email	34 (8.4%)	47 (6.5%)
Text Message	165 (40.7%)	314 (43.1%)
Phone call	199 (49.1%)	355 (48.8%)
Others[5]	7 (1.7%)	12 (1.6%)
**What operating time would you recommend for patients to access the smart lockers?**		
Morning (6 am – 12 noon)	77 (19.0%)	166 (22.8%)
Afternoon (12 noon – 4 pm)	43 (10.6%)	89 (12.2%)
Evening (4 pm – 10 pm)	20 (4.9%)	90 (12.4%)
24-hour access	265 (65.4%)	383 (52.6%)

Others[1]: Asthmatic, Surgical cases, Malaria, Hepatitis with stroke
Others[2]: Unspecified
Others[3] During the clinic visit, Roleplay, visual orientation, media service during clinic visit, home visit HCW, group discussion.
Others[4]: Any convenient location, Bank, Post office
Others[5]: Religious announcements, cluster collectors, disseminating information to other patients, face-to-face, home visits.

Table 2 reveals the participants' preferences for using smart lockers in dispensing chronic disease medication in Nigeria. Regarding the most suitable population to use the smart lockers, most participants responded that patients with HIV (HCWs – 43.2%, Patients – 45.9%) between the ages of 18 – 40 (HCWs – 81.0%, Patients – 70.5%) would benefit the most from using the smart lockers for dispensing their medication. The thoughts of HCWs and Patients align on the best way to orient them on using the smart lockers. The majority (HCWs – 53.8% and Patients – 58.1%) believed that one-on-one counselling by a healthcare provider should be used to orient the patients on using the smart lockers. Similarly, 71.6% of the patients agreed with the HCWs (68.9%) that smart lockers should be stationed at the hospital or clinic with 24-hour access (HCWs – 65.4% and patients – 52.6%). The majority (HCWs – 49.1% and Patients – 48.8%) also agreed that a phone call is the most preferred way of notifying patients of drug pick-up. Most HCWs (64.7%)

~~recommended pharmacists or other pharmacy staff as the best medical personnel to load the drugs into the smart lockers (Table 3).~~

Table 2~~3~~. The most suitable Personnel to load the drugs into the Smart Lockers in 5 states in Nigeria.

Personnel	HCWs, n = 405
HIV Case Manager	57 (14.1%)
Monitoring and Evaluation (M&E staff)	6 (1.5%)
Nurse	7 (1.7%)
Pharmacist or other pharmacy staff	262 (64.7%)
Specific staff employed for this sole purpose	66 (16.3%)
Others	7 (1.7%)

Others: Trained personnel and staff

Among healthcare workers (HCW), Table 2~~3~~ revealed that the most preferred personnel to load drugs into the smart lockers are pharmacists or other pharmacy staff (64.7%) against the Monitoring and evaluation (M&E) staff who had the lowest responses rate of (1.5%). Specific staff employed for this sole purpose was the next most preferred personnel to load drugs into smart lockers and HIV case managers had response rates of (16.3%) and (14.1%) respectively compared to Nurses and other professions who had lower response at (1.7%).

Table 3~~4~~. Preferences for the use of smart lockers in dispensing chronic disease medications in 5 states in Nigeria.

Variables	HCWs, n = 405	Patients, n = 728	p - value
**How might patient orientation be conducted on the use of the smart lockers?**			
Through audio-visuals	93 (23.0%)	147 (20.2%)	p = 0.25
Through printed material	91 (22.5%)	146 (20.1%)	
One-on-One counseling by Healthcare Provider	218 (53.8%)	423 (58.1%)	
Others[1]	3 (0.7%)	12 (1.6%)	
**What would be the best location for placing the smart lockers for access to patients?**			
Bus stop	2 (0.5%)	17 (2.3%)	$p < 0.05$
Community pharmacy	102 (25.2%)	139 (19.1%)	
Hospital or Clinic	279 (68.9%)	521 (71.6%)	
Police station	5 (1.2%)	6 (0.8%)	
Religious institution	9 (2.2%)	34 (4.7%)	
Others[2]	8 (2.0%)	11 (1.5%)	
**How best might patients receive notification of drug dispensing?**			

Formatted: Not Highlight

Email	34 (8.4%)	47 (6.5%)	$p = 0.63$
Text Message	165 (40.7%)	314 (43.1%)	
Phone call	199 (49.1%)	355 (48.8%)	
Others[3]	7 (1.7%)	12 (1.6%)	
**What operating time would you recommend for patients to access the smart lockers?**			
Morning (6 am – 12 noon)	77 (19.0%)	166 (22.8%)	$p < 0.05$
Afternoon (12 noon – 4 pm)	43 (10.6%)	89 (12.2%)	
Evening (4 pm – 10 pm)	20 (4.9%)	90 (12.4%)	
24-hour access	265 (65.4%)	383 (52.6%)	
**What patients that might benefit from using the smart lockers to collect their medication ~~non~~**			
Patients with HIV	175 (43.2%)	334 (45.9%)	$p < 0.05$
Patients with Cancer	41 (10.1%)	71 (9.8%)	
Patients with Diabetes	49 (12.1%)	81 (11.1%)	
Patients with Hypertension	47 (11.6%)	95 (13.0%)	
Patients with Tuberculosis	90 (22.2%)	106 (14.6%)	
Others[4]	3 (0.7%)	41 (5.7%)	
**For which of these age groups can the smart lockers be used in dispensing medication?**			
1 – 12 years	1 (0.2%)	8 (1.1%)	$p < 0.05$
13 – 17 years	5 (1.2%)	14 (1.9%)	
18 – 40 years	328 (81.0%)	513 (70.5%)	
41 – 60 years	55 (13.6%)	128 (17.6%)	
Above 60 years	16 (4.0%)	24 (3.3%)	
Others[5]	0 (0.0%)	41 (5.6%)	

Others[1]: Unspecified.
Other[2]: Any convenient location, Bank, Post office.
Others[3]: Religious announcements, cluster collectors, disseminating information to other patients, face-to-face, home visits.

Other[4]: Asthmatic, Surgical cases, Malaria, Hepatitis with stroke
Others[5]: Unspecified

Formatted: Superscript

Formatted: Superscript

The preference for one-on-one counselling as the best method of orientating patients on using smart lockers for dispensing chronic disease medication was higher in both the HWCs (53.8%) and the patients (58.1%), which signifies a preference for the use of smart lockers as a method for dispensing chronic disease medication, and this was no significant difference between the HCWs and the patients ($p = 0.25$). Most participants preferred a hospital or clinic as the secure and easily accessible location for the smart lockers. The preference was higher amongst patients (71.6%) than HWCs (68.9%). This was statistically significant with ($p < 0.05$).

Regarding the method of notification for drug delivery, the majority of HCWs (49.1%) and patients (48.8%) preferred using a phone call to notify patients when their drugs were dispensed into the smart lockers. There was no significant difference between (HCW and patients) ($p=0.63$).

There was a significant difference between the HCWs and Patients as both the HCWs (65.4%) and the patients (52.6%) accepted and preferred 24-hour access for the pick-up of drugs from the smart lockers with ($p<0.05$). Regarding the most suitable population to use the smart lockers, most participants responded that patients with HIV (HCWs – 43.2%, Patients – 45.9%) between the ages of 18 – 40 (HCWs – 81.0%, Patients – 70.5%) would benefit the most from using the smart lockers for dispensing their medication (Table 3).

(Table 5).

Discussion

The study was conducted to exploreexplored the potential benefits of implementing smart lockers for dispensing chronic disease medication in Nigeria. Specifically, it aimed to address the burden

on healthcare services and the lack of previous research on this topic in Nigeria. According to ~~the findings of our survey~~our survey findings, smart lockers can be strategically placed close to a secure clinic or hospital, allowing patients in the surrounding area 24-hour access to pick up their prescription drugs. Medication can be administered by calling the patients. Patients, or end users, need to be trained on how to use the smart lockers; getting one-on-one counselling from their doctors is the best course of action. Our study explored the preferences for ~~the use of~~using smart lockers for dispensing chronic disease medication, focusing on 5 states in Nigeria. According to Angell and others [~~24~~23], the quality of healthcare systems in low-middle-income countries like Nigeria is poor. This contributes to the increase in the burden of illnesses and global health costs. There is an increasing focus on strengthening health systems by investing in improving the quality of health services and creating equitable access to high-quality, people-centered care in building trust in the health system. To achieve these goals, the perspectives and preferences of patients and HCWs must be considered [24].

The overwhelming responses stemming from patients and HCWs regarding the populations best suited to use the smart lockers for chronic disease dispensing indicates that persons living with HIV between 18 and 40 years are the most suitable populations to use the smart lockers. This is likely due to the prevalence of HIV in Nigeria, with the highest HIV prevalence rates recorded among women between the ages of 25 and 34 [25, 26]. This may also be because most respondents in the survey were between the ages of 18 and 40. Additionally, decentralized care, such as community-based drug delivery and ART refills from community pharmacies, is popular in managing diseases such as HIV and Tuberculosis in Nigeria, unlike other chronic diseases [27, 28]. This would indicate a lesser resistance to adopting smart lockers as another model for decentralized care. However, the incidence of other chronic diseases, such as diabetes,

hypertension, and stroke, is higher in older populations aged 36 - 60 and above 60 years [29, 30]. There is increasing advocacy for improving person-centred care for older adults with chronic illnesses by transferring care from healthcare facilities to home and community-based models [31].

While most respondents advocate placing the lockers within a health institution such as a hospital, clinic, or community pharmacy, very few were open to using other locations such as bus stops, religious institutions, and police stations. This may be due to concerns relating to safety, privacy, and stigmatization [32]. Literature suggests that the rates of stigmatization differ based on the health condition, with persons suffering from HIV, Tuberculosis, and mental health conditions being among the most severely affected by stigmatization. These factors are often linked to a lack of awareness, beliefs, and institutionalized practices within healthcare facilities and communities [33, 34]. Decentralizing care and bringing the lockers to non-health-related institutions further expose patients to stigmatization and lack of privacy risks.

The patients and HCWs reported that they preferred smart lockers in a hospital or clinic. The sentiments behind this preference may be similar to those reported by Peng and others [35], who found that patients living with chronic diseases preferred to access care through primary healthcare (PHC) services, provided that the distance to the service provider was short and that they had access to capable HCWs. The use of designated areas in the community close to the patients, such as religious institutions or retail pharmacies for drug pick-ups for chronic non-communicable diseases, is reported to limit the patient's need for physician consultation to issues such as an error in prescription, delay in delivery and adverse drug reactions [36].

Results of our study also indicated that one-on-one counseling would be the most effective method of orientating patients using smart lockers. This may be linked to factors such as the literacy level of patients and their comprehension of health-related information disseminated through mediums

such as audio-visual tools and printed materials. Most patients in our study reported having completed a secondary school education (40.5%). Patient literacy has been suggested to influence individuals' health-seeking attitudes, impacting their ability to read, understand and act upon health-related information [37]. Additionally, many patients rely heavily on the opinions of their HCWs and are more likely to adopt treatment measures that a trusted physician suggests. This is especially applicable in the case of chronically ill persons who require ongoing care and have developed a strong relationship with their HCW over time [38, ~~39~~]. Having one-on-one contact with a Healthcare provider may overcome the barriers to understanding information about using smart lockers and increase their likelihood of adopting smart lockers for collecting their medication. A study by Donelan and others [~~40~~39] indicates that healthcare services should be made available to patients at any time of the day, considering the technological advancement the world is experiencing. Telemedicine affords a patient access to the physician without time restrictions and not necessarily having to go into the hospital [~~41~~40]. This points to the agreement found in this study from HCW and patients' responses to access time to smart lockers. Most patients and HCWs have the same line of thought that smart lockers for drug dispensing should be placed at the hospitals or clinics and made operational for 24 hours for easy access.

Furthermore, the usage of questionnaires may have resulted in self-selection bias, as respondents may have an essentially distinctive characteristic that affected their decision to prefer or not to prefer the use of smart lockers.

Due to the characteristics of the hospitals chosen and the fact that the HIV program provides the most extensive organized chronic care programs in those locations, the study has some limitations that may limit the generalizability of the findings to other chronic diseases.

In our study, most respondents opined that a phone call would be the best method for notifying patients when their drugs are dispensed. The use of smartphones has been reported to enhance patient drug adherence. Short Message Service (SMS) and synchronized data transmission applications enable healthcare providers to send reminders and keep up with patients' drug adherence [~~42~~41]. However, the ownership of mobile phones and smartphones in Nigeria is limited. Limited network access in certain areas may also hinder the quick and seamless transmission of electronic mail and SMS [~~43~~42]. This may be the reason why most of the respondents prefer receiving a phone call notification when their drugs are dispensed. This would be easier to track and ensure that the patients are adequately informed when their prescriptions are dispensed.

Conclusion

This is probably the first study assessing the preferences for using smart lockers in dispensing chronic disease medication among HCWs and persons living with chronic illnesses in Nigeria. The findings suggest that smart lockers may best serve PLHIV between the ages of 18 and 40 and this technology (Smart lockers) may be placed within a hospital or clinic that is within reach of patients in the community. In terms of engagement and operationality, enabling 24-hour access to the smart locker for drug pick-up and using phone calls to notify patients when their drugs are dispensed would be best suited for dispensing patient medication and the best method for orientating patients on the use of smart lockers may be one-on-one counseling from their healthcare providers. Additional research may be conducted to develop models for using smart lockers for dispensing chronic disease medications to other populations of chronically ill persons in Nigeria.

Acknowledgement

We would like to thank all those who contributed in one way or another to the collection and analysis of this data.To everyone on our team who contributed to this research, we thank you immensely for your extraordinary contributions and for putting in the effort to support science. You are deeply appreciated. A special thanks to all our partners who provided resource support, your contributions did not go unnoticed. Thank you for helping us succeed in this task.

Funding:

The study was funded by Georgetown University Medical Center (GUMC), Dean of Research. However, the funder did not play any role in the research.

S1 Table: Demographics of the Participants of Patients with Chronic Diseases and Health Care Workers in 5 States in Nigeria

S2 Table: The most suitable personnel to load the drugs into the smart lockers in 5 States in Nigeria.

S3 Table: Preferences for the use of smart lockers in dispensing chronic disease medications in 5 States in Nigeria

S1 Appendix: Distribution of states and facilities

S2 Appendix: Supporting data.

References

1. CDC (2021). National Center for Chronic Disease Prevention and Health Promotion. About Chronic Diseases. Retrieved 25 March 2022 from https://www.cdc.gov/chronicdisease/about/index.htm.
2. Anderson, E., & Durstine, J. L. (2019). Physical activity, exercise, and chronic diseases: A brief review. *Sports Medicine and Health Science*, *1*(1), 3-10.

3. Kunnumakkara, A. B., Sailo, B. L., Banik, K., Harsha, C., Prasad, S., Gupta, S. C., et al. (2018). Chronic diseases, inflammation, and spices: how are they linked? *Journal of translational medicine*, *16*(1), 14. https://doi.org/10.1186/s12967-018-1381-2.

4. World Health Organisation (2020). WHO reveals leading causes of death and disability worldwide: 2000-2019. Retrieved 25 March 2020 from https://www.who.int/news/item/09-12-2020-who-reveals-leading-causes-of-death-and-disability-worldwide-2000-2019.

5. World Health Organization (2018). Noncommunicable diseases country profiles 2018. Retrieved 25 March 2022 from https://apps.who.int/iris/handle/10665/274512.

6. Adesina, M.A., Oladele, R.I., Olufadewa, I.I., Onothoja, O.F., Oladipo, D.R., Iyiola, O.P., et al. (2021), "Addressing the high burden of noncommunicable diseases in Nigeria: a commentary", *Journal of Health Research*, Vol. 35 No. 5, pp. 457-462. https://doi.org/10.1108/JHR-02-2020-0039.

6. World Health Organization (2018).Global Health Observatory Data; Premature NCD deaths.Available from: https://www.who.int/gho/ncd/mortality_morbidity/ncd_premature_text/en/.

7. Sav, A., Salehi, A., Mair, F.S. *et al.* Measuring the burden of treatment for chronic disease: implications of a scoping review of the literature. *BMC Med Res Methodol* 17, 140 (2017). https://doi.org/10.1186/s12874-017-0411-8.

8. Hajat, C., & Stein, E. (2018). The global burden of multiple chronic conditions: A narrative review. *Preventive medicine reports*, *12*, 284–293. https://doi.org/10.1016/j.pmedr.2018.10.008

9. Faronbi, J. O., Ademuyiwa, I. Y., & Olaogun, A. A. (2020). Patterns of chronic illness among older patients attending a university hospital in Nigeria. *Ghana medical journal*, *54*(1), 42–47. https://doi.org/10.4314/gmj.v54i1.7.

Formatted: List Paragraph, Left, Add space between paragraphs of the same style, Line spacing: single

Formatted: Underline, Font color: Blue, Expanded by 0.85 pt, Pattern: Clear

10. De Villiers, K. Bridging the health inequality gap: an examination of South Africa's social innovation in health landscape. *Infect Dis Poverty* 10, 19 (2021). https://doi.org/10.1186/s40249-021-00804-9.

11. Idris, I.O., Oguntade, A.S., Mensah, E.A. *et al.* Prevalence of non-communicable diseases and its risk factors among Ijegun-Isheri Osun residents in Lagos State, Nigeria: a community based cross-sectional study. *BMC Public Health* 20, 1258 (2020). https://doi.org/10.1186/s12889-020-09349-2.

12. Büssing, A., Hübner, J., Walter, S., Gießler, W., & Büntzel, J. (2020). Tumor patients perceived changes of specific attitudes, perceptions, and behaviors due to the COVID-19 pandemic and its relation to reduced wellbeing. *Frontiers in psychiatry*, 883.

13. Jiang, Ch., Zhu, F. & Qin, (2020). Tt. Relationships between Chronic Diseases and Depression among Middle-aged and Elderly People in China: A Prospective Study from CHARLS. *CURR MED SCI* 40, 858–870 (2020). https://doi.org/10.1007/s11596-020-2270-5.

14. Yakubu, K., Malan, Z., Colon-Gonzalez, M. C., & Mash, B. (2018). Perceptions about family-centred care among adult patients with chronic diseases at a general outpatient clinic in Nigeria. *African journal of primary health care & family medicine*, *10*(1), e1–e11. https://doi.org/10.4102/phcfm.v10i1.1739.

15. Yusuff KB, Abd' Wassi Sanni. Itinerant vending of medicines inside buses in Nigeria: vending strategies, dominant themes and medicine-related information provided. PubMed. 2011 Jul

~~15.~~16. Mash, R., Christian, C., & Chigwanda, R. V. (2021). Alternative mechanisms for delivery of medication in South Africa: A scoping review. *South African family practice: official journal of the South African Academy of Family Practice/Primary Care*, *63*(1), e1–e8. https://doi.org/10.4102/safp.v63i1.5274.

~~16. Refaningati, T., Nahry, Tankdung, W. S. E. and Kusuma, A. (2020). Analysis of characteristics and efficiency of smart locker system (Case study: Jabodetabek). Evergreen, 7(1), 111–117. doi: 10.5109/2740966.~~

~~17. Wang, S. T., Li, M. H., & Lien, C. C. (2021). Analysis of the Optimal Application of Blockchain-Based Smart Lockers in the Logistics Industry Based on FFD-SAGA and Grey Decision-Making. *Symmetry*, *13*(2), 329. https://doi.org/10.3390/sym13020329.~~

~~18.~~17. Kiousis, V., Nathanail, E., Karakikes, I. (2019). Assessing Traffic and Environmental Impacts of Smart Lockers Logistics Measure in a Medium-Sized Municipality of Athens. In: Nathanail, E., Karakikes, I. (eds) Data Analytics: Paving the Way to Sustainable Urban Mobility. CSUM 2018. Advances in Intelligent Systems and Computing, vol 879. Springer, Cham. https://doi.org/10.1007/978-3-030-02305-8_74.

~~19.~~18. UNAIDS (2018). South Africa tests ATMs for medicine. Retrieved 28 March 2022 fromhttps://www.unaids.org/en/resources/presscentre/featurestories/2018/may/south-africa-tests-atms-for-medicine.

~~20.~~19. Asakitikpi, A. E. (2019). Chapter: Healthcare Coverage and Affordability in Nigeria: An Alternative Model to Equitable Healthcare Delivery. DOI: 10.5772/intechopen.85978

~~21.~~20. McKing, I. Ifunaya, C. (2021) Improving access, quality, and efficiency in health care delivery in Nigeria: a perspective. PAMJ - One Health. 2021; 5:3. doi: 10.11604/pamj-oh.2021.5.3.28204.

~~22.~~21. Ephraim-Emmanuel B, Adigwe A, Oyeghe R, Ogaji D (2018) 2875. Quality of health care in Nigeria: a myth or a reality. *International Journal of Research in Medical Science*- DOI - 10.18203/2320-6012.ijrms20183621.

~~23.~~22. Adebayo, O., Labiran, A., Emerenini, F. C. and Omoruyi, L. (2016). Health Workforce for 2016-2030: Will Nigeria have enough? International Journal of Innovative Healthcare Research. 4(1), 9-16.

~~24.~~23. Angell, B., Sanuade, O., Adetifa, I.M.O., Okeke, I.N., Adamu, A.L., Aliyu, M.H., et al. (2022). Population health outcomes in Nigeria compared with other west African countries, 1998–2019: a systematic analysis for the Global Burden of Disease Study. The Lancet, [online] 399(10330), pp.1117–1129. doi:https://doi.org/10.1016/S0140-6736(21)02722-7.

~~25.~~24. Institute for Health Metrics and Evaluation (IHME) (2020). GBD Compare Data Visualization. Seattle, WA: IHME, University of Washington, 2020. Retrieved 6 April 2022 from http://vizhub.healthdata.org/gbd-compare.

~~26.~~25. Federal Ministry of Health, Nigeria. Nigeria HIV/AIDS Indicator and Impact Survey (NAIIS) 2018: Technical Report. Abuja, Nigeria. October 2019. Accessed 11 April 2022 from http://ciheb.org/media/SOM/Microsites/CIHEB/documents/NAIIS-Report-2018.pdf

~~27.~~26. Rabkin, M., Strauss, M., Mantell, J. E., Mapingure, M., Masvawure, T. B., Lamb, M. R., et al. (2020). Optimizing differentiated treatment models for people living with HIV in urban Zimbabwe: Findings from a mixed methods study. *PloS one*, *15*(1), e0228148. https://doi.org/10.1371/journal.pone.0228148

~~28.~~27. Magadzire, B.P., Marchal, B. & Ward, K. (2016). Novel models to improve access to medicines for chronic diseases in South Africa: an analysis of stakeholder perspectives on community-based distribution models. *Journal of Pharmaceutical Policy and Practice,* **9,** 28 https://doi.org/10.1186/s40545-016-0082-6

~~29.~~28. Muthelo, L., Nemagumoni, T., Mothiba, T.M., Phukubje, A.T. and Mabila, L.N. (2020). Experiences of Professional Nurses Regarding the Implementation of a Central Chronic

Medicine Dispensing and Distribution Program at Primary Health Care Facilities in South Africa. The Open Public Health Journal, 13(1), pp.477–483. doi:https://doi.org/10.2174/1874944502013010477.

~~30.~~29. Zhao, J., Yan, C., Han, D., Wu, Y., Liao, H., Ma, Y., et al. (2022). Inequity in the healthcare utilization among latent classes of elderly people with chronic diseases and decomposition analysis in China. BMC Geriatrics, 22(1). doi:https://doi.org/10.1186/s12877-022-03538-x.

~~31.~~30. Nolte, E., Merkur, S., Anders Anell and European Observatory On Health Systems And Policies (2020). Achieving person-centred health systems : evidence, strategies and challenges. Cambridge ; New York, Ny: Cambridge University Press.

~~32.~~31. Dong R, Leung C, Naert MN, Naanyu V, Kiptoo P, Matelong W, et al. (2021) Chronic disease stigma, skepticism of the health system, and socio-economic fragility: Qualitative assessment of factors impacting receptiveness to group medical visits and microfinance for non-communicable disease care in rural Kenya. *PLoS ONE* 16(6): e0248496. https://doi.org/10.1371/journal.pone.0248496

~~33.~~32. Idemudia, E. S., Olasupo, M. O., & Modibo, M. W. (2018). Stigma and chronic illness: A comparative study of people living with HIV and/or AIDS and people living with hypertension in Limpopo Province, South Africa. *Curationis*, *41*(1), e1–e5. https://doi.org/10.4102/curationis.v41i1.1879

~~34.~~33. Nyblade, L., Stockton, M.A., Giger, K., Bond, V., Ekstrand, M. L., Mc Lean, R., et al. (2019). Stigma in health facilities: why it matters and how we can change it. *BMC Med* **17,** 25. https://doi.org/10.1186/s12916-019-1256-2

~~35.~~34. Peng Y, Jiang M, Shen X, Li X, Jia E, Xiong J. (2020). Preferences for Primary Healthcare Services Among Older Adults with Chronic Disease: A Discrete Choice Experiment. Patient Prefer Adherence;14:1625-1637. Doi: https://doi.org/10.2147/PPA.S265093

~~36.~~35. Krzyzaniak, N., Bajorek, B. A global perspective of the roles of the pharmacist in the NICU (2017). *International Journal of Pharmacy Practice*, Volume 25, Issue 2, April 2017, Pages 107–120, https://doi.org/10.1111/ijpp.12284

~~37.~~36. Grilo, A. M. , Santos, M. C. d. , Isabel Gomes, A., & Rita, J. S. (2017). Promoting Patient-Centered Care in Chronic Disease. In (Ed.), Patient Centered Medicine. IntechOpen. https://doi.org/10.5772/67380

~~38.~~37. Coventry, P. A., Fisher, L., Kenning, C., Bee, P., & Bower, P. (2014). Capacity, responsibility, and motivation: a critical qualitative evaluation of patient and practitioner views about barriers to self-management in people with multimorbidity. *BMC health services research*, *14*, 536. https://doi.org/10.1186/s12913-014-0536-y

~~39.~~38. Pritchard, D. E., Moeckel, F., Villa, M. S., Housman, L. T., McCarty, C. A., and McLeod, H. L. (2017). Strategies for integrating personalized medicine into healthcare practice. *Personalized Medicine*, 14,2, 141-152. Doi: 10.2217/PME-2016-0064

~~40.~~39. Donelan, K., Barreto, E. A., Sossong, S., Michael, C., Estrada, J. J., Cohen, A. B., et al. (2019). Patient and clinician experiences with telehealth for patient follow-up care. *The American journal of managed care*, *25*(1), 40–44.

~~41.~~40. Reed, M. E., Huang, J., Graetz, I., Lee, C., Muelly, E., Kennedy, C., et al. (2020). Patient Characteristics Associated With Choosing a Telemedicine Visit vs Office Visit With the Same Primary Care Clinicians. *JAMA network open*, *3*(6), e205873. https://doi.org/10.1001/jamanetworkopen.2020.5873

~~42.~~41. Al-Hassany L, Kloosterboer SM, Dierckx B, Koch BCP (2019). Assessing methods of measuring medication adherence in chronically ill children–a narrative review. *Patient Preference and Adherence*; 13:1175-1189. Doi: https://doi.org/10.2147/PPA.S200058

~~43.~~42. Ivan Forenbacher, Siniša Husnjak, Ivan Cvitić, Ivan Jovović, (2019). Determinants of mobile phone ownership in Nigeria. *Telecommunications Policy*,43, 7, 101812. Doi: https://doi.org/10.1016/j.telpol.2019.03.00

Dear Dr Orvalho Augusto, Reviewer 2 and Reviewer 3,

We express our gratitude for your valuable feedback and the chance to submit a revised draft of our manuscript, now titled Patients and Healthcare Workers' Preferences for Using Smart Lockers in Dispensing Chronic Disease Medication in Nigeria: Findings from a Descriptive Cross-Sectional Study. The meticulous attention to detail, as well as the time and resources dedicated to offering constructive comments and reviews, is sincerely appreciated. We have diligently incorporated changes to address the majority of the suggestions provided, ensuring that we contextualize some of the comments within the paper. Your input has significantly enhanced the quality and comprehensiveness of our work.

The following are the responses to the journal requirements:

1. The manuscript has been adjusted to reflect the PLOS One filing system requirement.
2. The PLOS questionnaire on inclusivity in global research does not apply because the research was conducted locally.
3. The acronym GUMC has been expanded accordingly.
4. Captions have been included at the end of the document.

Reviewer 1 (Orvalho Augusto): Thank you for your response, the subdivisions in this section have been merged. Lines 131 to 133 and lines 135 to 136 have been adjusted.

Reviewer 1 (Orvalho Augusto): Thank you for your response, the 50% expected response point is included in the sample size determination.

Reviewer 1 (Orvalho Augusto): We are appreciative of your feedback. The statement has been corrected to reflect the WHO definition of premature mortality.

Reviewer 1 (Orvalho Augusto): Thank you for pointing this out in line 195. It has been corrected.

Reviewer 1 (Orvalho Augusto): Thank you for your response. During the study, we considered the HCW as a unit providing care to patients living with chronic diseases, therefore, we did not collect the cadre of the participating HCWs, we only sampled their opinion on the suitable personnel for loading drugs into the smart lockers.

Table 2 and 4 have been harmonized.

Reviewer 2: Thank you for your suggestion on the topic. the title has been adjusted to reflect "Patients and Healthcare Workers' Preferences for Using Smart Lockers in Dispensing Chronic Disease Medication in Nigeria—findings from a descriptive cross-sectional study"

Reviewer 2: Thank you for your comments on this, we asked both the HCWs and patients same sets of questions on the preference of smart lockers in order to get an insight from HCWs who provide services and patients who receive services.

In addition HCW participants were exclusively asked on the suitable personnel for loading the smart lockers. Adjust lastly to reflect changes

Reviewer 2: Thank you for your question, the patients with these chronic diseases were examined.

728 (Table1)

Reviewer 2: Thank you for your feedback, this adjustment has been made to reflect 5 states as conducted in the study.

Thank you for pointing this out. "Participants have been included after 1,133 in line 30 in the manuscript"

Reviewer 2: Thank you and this is noted. Absolute numbers have been included in lines 31 and 32

Reviewer 2: The comment is appreciated, the statement on line 32 has been re-worded as findings.

Reviewer 2: Thank you for your response, it was 1,180 responses. The responses were received through electronic mails, social media platforms such as WhatsApp, LinkedIn, Instagram, and Facebook.

Reviewer 2: Thank you for your feedback on this, Line 91-97 have been removed and the sectionized problem statement and justification have been consolidated into the introduction.

Reviewer 2: Your response on this is deeply appreciated, the objectives statement has been adjusted to reflect just the objective and the numbering has been removed.

Reviewer 2: Thank you for your comments on this. The survey was conducted within the sites of the Implementing partners (IPs) not exclusively to the patients and healthcare workers of the Implementing Partners. Secondly, both the HCWs and patients answered the same sets of

questions. Additionally, HCW participants were exclusively asked on the suitable personnel for loading the smart lockers.

Reviewer 2: Thank you for noting this comment, the participants were people above the age of 18 and children were not involved.

Reviewer 2: Thank you for pointing this out the lines 162-164inclusive of the study population have been merged into one statement.

Reviewer 2: Thank you for your comments on this, the survey was conducted within the sites of the Implementing partners (IPs) not exclusively to the patients and healthcare workers of the Implementing Partners.

Reviewer 2: This comment is appreciated. The ethical statement has been adjusted including adjustment to line 202.

Reviewer 2: Thank you for this comment on the titles of the tables. The titles of the tables have been adjusted as suggested.

Reviewer 2: Thank you, this is noted. The titles of the tables have been adjusted. The age band in this category was 18-35 years of age not limited to 18 years.

Reviewer 2: The basis for the selection of healthcare workers was the healthcare workforce that manages chronic disease at the clinics.

Reviewer 2: Thank you for your comment on what HCW/patients might benefit from using smart lockers, this has been addressed.

Reviewer 2: Thank you for noting this in this comment, tables 2 and 4 have been consolidated.

Reviewer 2: Thank you for your comments. The difference in lines 280-282 is addressed.

Reviewer 2: We are appreciative of your comment about the discussion, our discussion section has been adjusted accordingly.

Reviewer 3: Thank you. Further information has been provided in the background concerning traditional method of dispensing drugs.

Reviewer 3: Thank you for your comment, the number of states has been adjusted accordingly.

Reviewer 3: Thank you for your response. The participants were selected or identified through implementing partners in the country. These partners manage healthcare facilities within the 5 States captured in the study.

Reviewer 3: Thank you for your comment. This is noted.

Reviewer 3: Thank you for this comment. This is noted and adjusted.

Reviewer 3: Thank you for your comment on the questionnaire. The questionnaire was self-administered through electronic mail, social media networking sites and groups, through platforms, such as WhatsApp, Facebook, Instagram, and LinkedIn. The smart lockers will be both manually and digitally operated.

Reviewer 3: Thank you for this comment on sharing the TIFF format for figures within the document, but currently, this is not applicable as there are tables in the document but no figures.

We look forward to hearing from you in due time regarding our submission and to responding to any further questions and comments you may have.

Sincerely,

Ibrahim Bola Gobir.

Response to reviewer 2 comments

Thank you for taking the time to review our manuscript titled "Patients and Healthcare Workers' Preferences for Using Smart Lockers in Accessing and Dispensing Chronic Disease Medication in Nigeria: Findings from a Descriptive Cross-Sectional Study" submitted to PLOS ONE We appreciate your insightful comments and constructive feedback, which have greatly contributed to improving the quality of our work.

Reviewer 2: Thank you for this response, The title has been revised as suggested.

Reviewer 2: Thank you for your comment. The word "Participants" has been included in the bracket.

Reviewer 2: We appreciate your feedback. The statement has been adjusted to sound more meaningful.

Reviewer 2: Your comment is appreciated, in the background of our study, the paragraphs have been adjusted.

Reviewer 2: Thank you for your comment. This adjustment was made during the previous revision and the subtitles merged into the background.

Reviewer 2: We are appreciative of your feedback. This adjustment was made during the previous revision and the sections were merged into the background.

Reviewer 2: Thank you for your comment. This adjustment to the methods was made during the previous revision.

Reviewer 2: Thank you for your response. The discussion session has been adjusted.

Reviewer 2: Thank you for your suggestion on the acknowledgement. It has been adopted.
