# Supplementary material for: Patients and healthcare workers’ preferences for using smart lockers in accessing and dispensing chronic disease medication in Nigeria: Findings from a descriptive cross-sectional study
Source: PLoS One. 2024 Jul 5;19(7):e0303625. doi: 10.1371/journal.pone.0303625 (PMC11226012; doi:10.1371/journal.pone.0303625)
Supplement: Attachment — Submitted filename: Response letter to reviewers on PLOS ONE 13_2_2024.docx [file pone.0303625.s007.docx]

Dear Dr Orvalho Augusto, Reviewer 2 and Reviewer 3,

We express our gratitude for your valuable feedback and the chance to submit a revised draft of our manuscript, now titled Patients and Healthcare Workers' Preferences for Using Smart Lockers in Dispensing Chronic Disease Medication in Nigeria: Findings from a Descriptive Cross-Sectional Study. The meticulous attention to detail, as well as the time and resources dedicated to offering constructive comments and reviews, is sincerely appreciated. We have diligently incorporated changes to address the majority of the suggestions provided, ensuring that we contextualize some of the comments within the paper. Your input has significantly enhanced the quality and comprehensiveness of our work.

The following are the responses to the journal requirements:

1. The manuscript has been adjusted to reflect the PLOS One filing system requirement.
2. The PLOS questionnaire on inclusivity in global research does not apply because the research was conducted locally.
3. The acronym GUMC has been expanded accordingly.
4. Captions have been included at the end of the document.

Reviewer 1 (Orvalho Augusto): Thank you for your response, the subdivisions in this section have been merged. Lines 131 to 133 and lines 135 to 136 have been adjusted.

Reviewer 1 (Orvalho Augusto): Thank you for your response, the 50% expected response point is included in the sample size determination.

Reviewer 1 (Orvalho Augusto): We are appreciative of your feedback. The statement has been corrected to reflect the WHO definition of premature mortality.

Reviewer 1 (Orvalho Augusto): Thank you for pointing this out in line 195. It has been corrected.

Reviewer 1 (Orvalho Augusto): Thank you for your response. During the study, we considered the HCW as a unit providing care to patients living with chronic diseases, therefore, we did not collect the cadre of the participating HCWs, we only sampled their opinion on the suitable personnel for loading drugs into the smart lockers.

Table 2 and 4 have been harmonized.

Reviewer 2: Thank you for your suggestion on the topic. the title has been adjusted to reflect “Patients and Healthcare Workers' Preferences for Using Smart Lockers in Dispensing Chronic Disease Medication in Nigeria—findings from a descriptive cross-sectional study”

Reviewer 2: Thank you for your comments on this, we asked both the HCWs and patients same sets of questions on the preference of smart lockers in order to get an insight from HCWs who provide services and patients who receive services.

In addition HCW participants were exclusively asked on the suitable personnel for loading the smart lockers. Adjust lastly to reflect changes

Reviewer 2: Thank you for your question, the patients with these chronic diseases were examined.

728 (Table1)

Reviewer 2: Thank you for your feedback, this adjustment has been made to reflect 5 states as conducted in the study.

Thank you for pointing this out. “Participants have been included after 1,133 in line 30 in the manuscript”

Reviewer 2: Thank you and this is noted. Absolute numbers have been included in lines 31 and 32

Reviewer 2: The comment is appreciated, the statement on line 32 has been re-worded as findings.

Reviewer 2: Thank you for your response, it was 1,180 responses. The responses were received through electronic mails, social media platforms such as WhatsApp, LinkedIn, Instagram, and Facebook.

Reviewer 2: Thank you for your feedback on this, Line 91-97 have been removed and the sectionized problem statement and justification have been consolidated into the introduction.

Reviewer 2: Your response on this is deeply appreciated, the objectives statement has been adjusted to reflect just the objective and the numbering has been removed.

Reviewer 2: Thank you for your comments on this. The survey was conducted within the sites of the Implementing partners (IPs) not exclusively to the patients and healthcare workers of the Implementing Partners. Secondly, both the HCWs and patients answered the same sets of questions. Additionally, HCW participants were exclusively asked on the suitable personnel for loading the smart lockers.

Reviewer 2: Thank you for noting this comment, the participants were people above the age of 18 and children were not involved.

Reviewer 2: Thank you for pointing this out the lines 162-164inclusive of the study population have been merged into one statement.

Reviewer 2: Thank you for your comments on this, the survey was conducted within the sites of the Implementing partners (IPs) not exclusively to the patients and healthcare workers of the Implementing Partners.

Reviewer 2: This comment is appreciated. The ethical statement has been adjusted including adjustment to line 202.

Reviewer 2: Thank you for this comment on the titles of the tables. The titles of the tables have been adjusted as suggested.

Reviewer 2: Thank you, this is noted. The titles of the tables have been adjusted. The age band in this category was 18-35 years of age not limited to 18 years.

Reviewer 2: The basis for the selection of healthcare workers was the healthcare workforce that manages chronic disease at the clinics.

Reviewer 2: Thank you for your comment on what HCW/patients might benefit from using smart lockers, this has been addressed.

Reviewer 2: Thank you for noting this in this comment, tables 2 and 4 have been consolidated.

Reviewer 2: Thank you for your comments. The difference in lines 280-282 is addressed.

Reviewer 2: We are appreciative of your comment about the discussion, our discussion section has been adjusted accordingly.

Reviewer 3: Thank you. Further information has been provided in the background concerning traditional method of dispensing drugs.

Reviewer 3: Thank you for your comment, the number of states has been adjusted accordingly.

Reviewer 3: Thank you for your response. The participants were selected or identified through implementing partners in the country. These partners manage healthcare facilities within the 5 States captured in the study.

Reviewer 3: Thank you for your comment. This is noted.

Reviewer 3: Thank you for this comment. This is noted and adjusted.

Reviewer 3: Thank you for your comment on the questionnaire. The questionnaire was self-administered through electronic mail, social media networking sites and groups, through platforms, such as WhatsApp, Facebook, Instagram, and LinkedIn. The smart lockers will be both manually and digitally operated.

Reviewer 3: Thank you for this comment on sharing the TIFF format for figures within the document, but currently, this is not applicable as there are tables in the document but no figures.

We look forward to hearing from you in due time regarding our submission and to responding to any further questions and comments you may have.

Sincerely,

Ibrahim Bola Gobir.
