# Supplementary material for: Patients and healthcare workers’ preferences for using smart lockers in accessing and dispensing chronic disease medication in Nigeria: Findings from a descriptive cross-sectional study
Source: PLoS One. 2024 Jul 5;19(7):e0303625. doi: 10.1371/journal.pone.0303625 (PMC11226012; doi:10.1371/journal.pone.0303625)
Supplement: Attachment — Submitted filename: Response Letter to Reviewers_Man 3_21st April 2024.docx [file pone.0303625.s010.docx]

Dear Dr Mohammed Shuaib,

Thank you for providing valuable feedback and allowing us to resubmit a revised manuscript draft titled "Patients and Healthcare Workers' Preferences for Using Smart Lockers in Accessing and Dispensing Chronic Disease Medication in Nigeria: Findings from a Descriptive Cross-Sectional Study." Your thorough review has undoubtedly enhanced the quality and clarity of our work. Below, we have provided a detailed response to each point raised, outlining the revisions made to strengthen the manuscript.

Thank you for your comment. A graphical representation has been provided to enhance the presentation of results for Tables 1, 2, and 3

Thank you for your comment. The paper has been proofread and readjusted to remove errors.

Thank you for your suggestion. No changes was made to the financial disclosure statement.

Your comment on the background is well appreciated. The background of the study has been streamlined as requested.

Your comment is well received. Lengthy sentences have been made shorter and concise as prescribed.

Thank you for your comment, existing gaps in literature regarding our study are captured in the latter of the background section on page 5

Thank you for your comment on the study design. Additional context to explain why the descriptive cross-sectional design has been captured in the method section on page 6, this is captured through an explanation of our study population.

Your comment is very well appreciated on the inclusion criteria. Additional context has been provided in the inclusion criteria captured on page 6

Thank you for your comment. An additional paragraph has been added in the method section describing the selection of participants. The sample size was for both healthcare workers and patients have been included in the section as well.

Your comment is very well appreciated and received. An additional statement has been provided under the paragraph for each variable analyzed during the study and the statistical test performed on page 7.

Thank you for your comment. We have explained in the methods section on page 8, the assumptions made during the data analysis and the justification for using Stata 15 for analysis.

Your comment is well received and appreciated. On page 15 emphasis on the lack of research on smart lockers and implementation has been included as a preamble to discussing our findings.

Thank you for your comment. The preference for smart lockers among persons living with HIV aged 18-40 among most suitable settings is included in line 261 – 268

Thank you for your observation. The practicality of one-on-one counselling for patient orientation is discussed on page 17 lines 291-309
